# Keratinocyte and Fibroblast Wound Healing In Vitro Is Repressed by Non-Optimal Conditions but the Reparative Potential Can Be Improved by Water-Filtered Infrared A

**DOI:** 10.3390/biomedicines9121802

**Published:** 2021-11-30

**Authors:** Cornelia Wiegand, Uta-Christina Hipler, Peter Elsner, Jörg Tittelbach

**Affiliations:** Department of Dermatology, University Hospital Jena, Erfurter Str. 35, 07743 Jena, Germany; christina.hipler@med.uni-jena.de (U.-C.H.); elsner@derma-jena.de (P.E.); joerg.tittelbach@med.uni-jena.de (J.T.)

**Keywords:** wound healing, scratch assay, keratinocyte, fibroblast, UV-B, wIRA (water-filtered infrared A), pro-inflammatory cytokines, heat shock protein, desmoglein, collagen

## Abstract

It is a general goal to improve wound healing, especially of chronic wounds. As light therapy has gained increasing attention, the positive influence on healing progression of water-filtered infrared A (wIRA), a special form of thermal radiation, has been investigated and compared to the detrimental effects of UV-B irradiation on wound closure in vitro. Models of keratinocyte and fibroblast scratches help to elucidate effects on epithelial and dermal healing. This study further used the simulation of non-optimal settings such as *S. aureus* infection, chronic inflammation, and anti-inflammatory conditions to determine how these affect scratch wound progression and whether wIRA treatment can improve healing. Gene expression analysis for cytokines (*IL1A*, *IL6*, *CXCL8*), growth (*TGFB1*, *PDGFC*) and transcription factors (*NFKB1*, *TP53*), heat shock proteins (*HSP90AA1*, *HSPA1A*, *HSPD1*), keratinocyte desmogleins (*DSG1*, *DSG3*), and fibroblast collagen (*COL1A1*, *COL3A1*) was performed. Keratinocyte and fibroblast wound healing under non-optimal conditions was found to be distinctly reduced in vitro. wIRA treatment could counteract the inflammatory response in infected keratinocytes as well as under chronic inflammatory conditions by decreasing pro-inflammatory cytokine gene expression and improve wound healing. In contrast, in the anti-inflammatory setting, wIRA radiation could re-initiate the acute inflammatory response necessary after injury to stimulate the regenerative processes and advance scratch closure.

## 1. Introduction

Wound healing is a complex process in which different epidermal and dermal cell types as well as leukocytes are involved and is coordinated by cytokines and growth factors [1]. The focus here was on fibroblasts, which are involved in the production of extracellular matrix and thus fill the wound with new tissue [2], and keratinocytes, which are involved in the inflammatory reaction and can ultimately close the injured area [3]. Prolonged inflammation delays wound closure by preventing the transition from degrading to proliferative processes [4]. Chronic wounds represent a major challenge in hospitals and health care settings. These wounds may remain non-healing due to a microbial infection [5,6] and continuous inflammatory conditions [7]. Moreover, anti-inflammatory conditions have been reported to affect wound healing processes [8,9,10].

Positive effects on wound healing by photobiomodulation through light therapy have been described [11], so it is of interest to investigate the effects of light treatment on cell viability and cell proliferation during wound healing. Recently, acceleration of healing processes after application of low-level laser therapy (LLLT) has been evaluated [12]. Red light (600–760 nm) was found to induce the migration of human epidermal stem cells [13], stimulate keratinocyte proliferation [14], and increase migration of fibroblasts isolated from diabetic patients [15]. Red or infrared light with less than 200 mW/cm^2^ irradiance and a wavelength range of 600–1000 nm was further reported to promote the skin repair process in experimental animal and human wounds [16,17,18,19]. This suggests beneficial roles for red light therapy in re-epithelialization and extracellular matrix formation during wound healing, probably by enhancing fibroblast migration and proliferation as well as collagen synthesis [20,21] and by modulating the timing and release of growth factors and cytokines [22].

Water-filtered infrared A (wIRA) is a special form of thermal radiation with high penetration capacity into the tissue with low thermal surface load. It is characterized by a deep-reaching thermal radiation with comparatively low heat-induced pain sensation on the skin surface in its typical irradiance levels between 60 and 120 mW/cm^2^ [23,24]. Studies have shown that it promotes the healing of acute and chronic wounds through thermal and non-thermal effects [25,26,27]. Indeed, it has been found that wIRA-irradiation evokes an increase in local temperature [26,27], oxygen partial pressure [27], and tissue blood flow in wounds [26]. These factors substantially support the healing process, especially in chronic wounds, where generally ischemic, hypoxic, and hypothermic conditions are found [28]. Additionally, wIRA has nonthermal effects, which are thought to directly stimulate cells and cellular structures by infrared radiation. These range from stimulation of wound repair [29] and cell protective effects [30] to target-oriented growth of neurons [31] and possible effects on pain receptors [27]. So far, Zöller et al. have reported on foreskin and keloid fibroblasts treated with convective heat and/or wIRA to assess their potential as treatment regimens for keloids and hypertrophic scars [32]. They found a temperature dependent induction of a spherical cell shape, a reduction in collagen type I synthesis, and decreased TGF-β1 secretion in the fibroblasts. No influence of wIRA-irradiation on MMP-1 was observed. However, wIRA-treatment restored original cell morphology in foreskin fibroblasts, and beyond that, collagen type I synthesis and TGF-β1 secretion in keloid fibroblasts [32]. Knels et al. evaluated wIRA for the treatment of oxidative stress in cells as it is found in elderly or diabetic patients [33]. Therefore, they exposed fibroblast cultures to glyoxal to induce glycation of proteins and lipids, mimicking the conditions of oxidative stress, and determined their rescue from apoptotic death by wIRA. They found that wIRA-irradiation diminished the effects of glyoxal-induced stress such as ROS production, translocation of phosphatidylserine, and DNA fragmentation, which are considered the main events of apoptosis, while being well tolerated by the cultured fibroblasts [33].

It is therefore of interest to further elucidate the cellular effects of wIRA in order to be able to use this effectively as a treatment option. The knowledge gained provides important information on the cellular influence of wIRA on the processes taking place in keratinocytes and fibroblasts during wound healing. Fibroblasts are involved in the production of extracellular matrix and are therefore essential to fill the wound with new tissue [2]. Keratinocytes are not only involved in the eventual closure of the injured site through epithelialization, but also take part directly in immunological processes and inflammatory reactions as non-professional immune cells [3,34,35]. One of the most studied in vitro models for wound healing is the mechanical damage to confluent cell layers (“scratch wound assay”) [36]. This method enables the direct measurement of cell migration and regeneration of the cell layer. HaCaT cells, primary epidermal keratinocytes, and primary dermal fibroblasts have already been successfully employed in the scratch wound assay [37]. Preliminary studies for wIRA-effects on wound closure in vitro have already shown distinct differences of a 10 min treatment of wounded HaCaT keratinocyte monolayers compared to the untreated control and heat treatment alone [38]. A further advantage of the scratch wound assay is that concentrations of growth factors and cytokines can be easily assessed by flow cytometry or ELISA techniques and gene transcript levels can be determined by real-time qPCR [39]. Furthermore, the milieu of scratch assays can be easily altered to reflect different wound conditions [40]. Here, cells were infected with *S. aureus* to mimic wound infection, primed with TNF-α and IFN-γ to establish chronic inflammatory conditions [41], and stimulated with IL-4 and IL-13 to yield a TH2-dominant, anti-inflammatory milieu [9,42].

In this study, keratinocyte and fibroblast wound healing in vitro under optimal and non-optimal conditions was investigated using cell scratch wound healing assays. Furthermore, gene expression analysis for pro-inflammatory cytokines (*IL1A*, *IL6*, *CXCL8*), growth factors (*TGFB1*, *PDGFC*), transcription factors (*NFKB1*, *TP53*), and heat shock proteins (*HSP90AA1*, *HSPA1A*, *HSPD1*) as well as desmogleins (*DSG1*, *DSG3*) in keratinocytes and collagen (*COL1A1*, *COL3A1*) in fibroblasts was performed to evaluate cellular responses to healing progression (Table 1) under optimal conditions and infection with *S. aureus* as well as after priming with TNF-α/IFN-γ (chronic inflammatory milieu) or IL-4/IL-13 (anti-inflammatory conditions). Moreover, scratches were treated with wIRA and heating *w*/*o* wIRA as the control to discriminate between thermal and non-thermal effects of wIRA [43,44] and scratches under optimal conditions were also irradiated with UV-B for comparison.

## 2. Materials and Methods

Keratinocyte and fibroblast culture: HaCaT cells (provided by Prof. Fusenig, Heidelberg, Germany) and normal human dermal fibroblasts (NHDF; Promocell, Heidelberg, Germany) were cultured in Dulbecco’s modified Eagle’s Medium (DMEM; BioConcept, Alschwil, Switzerland) supplemented with 1% antibiotic-antimycotic solution (BioConcept, Alschwil, Switzerland) and 10% fetal calf serum (FCS; PAN-Biotech, Aidenbach, Germany). The cells were cultured for seven days in 75-cm^2^ cell culture flasks (Greiner bio-one, Frickenhausen, Germany) at 37 °C and in a humidified atmosphere containing 5% CO_2_. For experiments, cells were harvested through trypsin-EDTA (Gibco Thermo Fisher Scientific, Waltham, MA, USA) treatment, seeded into 12-well plates (Greiner bio-one, Frickenhausen, Germany) at a density of 40,000 cells/cm^2^, and cultured for 48 h to confluence.

Scratch wound assay: Cell monolayers were scratched with a sterile pipette tip and washed with medium to remove any loose cells. Scratches were then treated with the light emitting devices and scratches kept in the incubator at 37 °C served as untreated controls. Cells were incubated for 1, 3, 6, 12, 24, and 48 h before staining with hematoxylin (Merck Millipore, Darmstadt, Germany) for the evaluation of scratch closure progression or gene expression analysis. Microscopic assessment of the scratches was carried out using a VHX 950F digital microscope (KEYENCE DEUTSCHLAND GmbH, Neu-Isenburg, Germany) and images were obtained. The scratch area was determined in mm^2^ using the VHX 950F software (v01., KEYENCE DEUTSCHLAND GmbH, Neu-Isenburg, Germany). From these results, scratch wound healing was calculated in [%] for each time point relative to the scratch area at 1 h. To test the scratch wound healing under non-optimal conditions, cell culture media were replaced by primed media 24 h before the experiment. Chronic inflammatory conditions were mimicked by the addition of 10 ng/mL TNF-α (7Bioscience, Neuenburg, Germany) and 5 ng/mL IFN-γ (7Bioscience, Neuenburg, Germany) while anti-inflammatory conditions were simulated using 50 ng/mL IL-4 (7Bioscience, Neuenburg, Germany) and 50 ng/mL IL-13 (7Bioscience, Neuenburg, Germany). To investigate wound healing under infection conditions, cells were infected with *Staphylococcus aureus* 1 h before the start of the experiments using 10^2^ cfu/mL in cell culture media. For preparation of bacteria, *S. aureus* ATCC 6538 (DSMZ, Braunschweig, Germany) colonies from Columbia agar plates (bioMerieux, Nürtingen, Germany) were suspended in Tryptic Soy broth (TSB, Oxoid, Dardilly, France) and cultivated for 24 h at 37 °C under vigorous shaking. The number of microbes in the solution was determined by serial dilution followed by plating on Columbia agar. After 24 h incubation at 37 °C, colonies were counted and microbial count (in CFU/mL) of the bacterial suspension was calculated.

Treatment with light emitting devices and controls: Treatments were performed in 12-well plates after replacing the culture medium with 800 µL PBS (phosphate buffered saline, Promocell, Heidelberg, Germany) to avoid desiccation of the cells. Samples were treated with wIRA (wIRA-irradiator type 750; Hydrosun^®^, Müllheim, Germany) at a distance of 33 cm for 20 min. Control experiments were included with a simulated increase in temperature of 8 degrees over 20 min as it may occur during a clinical application of wIRA [26] to distinguish between effects caused by the wIRA-treatment and pure warming of the sample. These samples were designated as heating *w*/*o* wIRA. Temperature profiles were recorded locally on the cells using thin thermocouples (type K, TC Direct, Mönchengladbach, Germany). In addition, tests were performed with UV-B irradiation (UV109; Waldmann Medizintechnik, Villingen-Schwenningen, Germany) at a distance of 16 cm for 20 min.

Gene expression analysis: After removal of the cell culture supernatants, human cells were lysed by adding RLT buffer (Qiagen, Hilden, Germany) containing 10 µL/mL β-mercaptoethanol and subsequently incubated for 3 min on ice and 3 min under shaking. Lysates were subsequently loaded to QIA Shredder spin columns (Qiagen, Hilden, Germany), and centrifuged at 4 °C and 10.000× *g* for 2 min. RNA purification was performed using the RNeasy^®^ Mini Kit and the QIAcube (Qiagen, Hilden, Germany). To avoid DNA contamination, genomic DNA was removed from the extracted RNA using the Ribonuclease assay DNase I, RNase Free (Thermo Fisher Scientific, Waltham, MA, USA). A single master mix prepared on ice contained 41 µL extracted RNA, 5 µL 10× Reaction Buffer (Mg^2+^), and 4 µL DNase I. The latter was activated at 37 °C for 30 min. Finally, EDTA was added as a chelating agent to stop the Mg^2+^-dependent nuclease activity of DNase I during 10 min incubation at 65 °C. RNA concentration was determined using the SPECTROstar^®^ Omega with a UV/Vis plate (BMG Labtech, Ortenberg, Germany) by measuring the OD at 260 nm, with the assumption that OD of 1.0 equals 40 ng/mL RNA. The purity of the RNA sample was evaluated using the ratio of the absorbance at 260 nm and 280 nm with a ratio’s threshold between 1.9 and 2.1. All samples fulfilled this requirement. Subsequently, absolute RNA amounts were adjusted to 60–400 ng in 10 µL. For reverse transcription, the High Capacity cDNA Reverse Transcription Kit by Applied Biosystems (Thermo Fisher Scientific, Waltham, MA, USA) was used following the manufacturer’s instructions. The PCR protocol ran with the Mastercycler^®^ gradient thermal cycler (Eppendorf) included primer annealing for 10 min at 25 °C, reverse transcription for 120 min at 37 °C, and termination for 5 min at 85 °C cDNA samples were stored at −80 °C until further use. After reverse transcription, cDNA was diluted respectively to obtain a final test concentration of 0.5 ng/mL. RTqPCR was performed for gene expression analyses using the QuantiNova™ SYBR Green PCR Kit (Qiagen, Hilden, Germany) following the manufacturer’s instructions. Briefly, master mix prepared on ice contained forward and reversed primers (test concentration each 0.5 µM) and cDNA or Yellow Template Dilution Buffer as no template control. Using the qTOWER3G (Analytik Jena AG, Thuringia, Germany), the real-time amplification protocol was set for polymerase heat activation at 95 °C for 3 min, and 40 cycles with three steps: denaturation at 95 °C for 5 s, annealing at 57 °C for 10 s, and elongation at 72 °C for 10 s. Signals were detected at λex/λem 470 nm/520 nm. Finally, a melting curve from 65 °C to 95 °C served as the amplicon control. All primer sequences or ordering IDs are listed in Table 2. Samples were measured in technical duplicates. Expression levels of target genes were normalized to the housekeeping gene *ACTB*.

Data analysis: Two independent experiments were executed and measurements were performed in duplicate. Transcription levels are presented as fold changes of the respective untreated control under optimal conditions at 1 h. All values are expressed as means ± SD (standard deviation). One-way analysis of variance was carried out to determine statistical significances (Microsoft^®^ Excel 2010). Differences were considered statistically significant at a level of *p* < 0.05. Asterisks [*] indicate significant differences in scratch wound healing or transcription level compared to 1 h (* *p* < 0.05, ** *p* < 0.01, *** *p* < 0.001). Hashtags [#] designate significant differences of scratch wound healing or transcription levels at the respective time point compared to the control under optimal conditions (# *p* < 0.05, ## *p* < 0.01, ### *p* < 0.001) while the paragraph character [§] shows significant differences between untreated and treated samples under non-optimal conditions at the respective time point (§ *p* < 0.05, §§ *p* < 0.001, §§§ *p* < 0.001). The plus sign [+] specifies significant differences between wIRA-treated samples and those receiving heating *w*/*o* wIRA under non-optimal conditions at the respective time point (+ *p* < 0.05, ++ *p* < 0.01, +++ *p* < 0.001).

## 3. Results

### 3.1. Scratch Wound Healing and Gene Expression Analysis over Time

**Figure 1:**
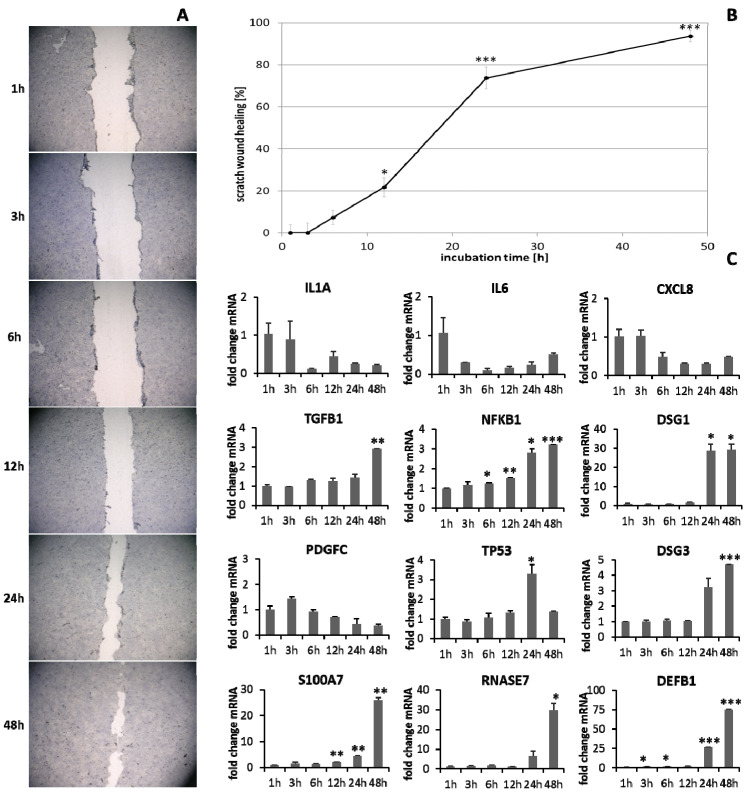
(**A**) Scratch wound healing of HaCaT keratinocytes under optimal conditions and (**B**) evaluation of scratch wound healing in [%]. (**C**) Gene expression profiles over time during wound healing. Asterisks [*] indicate significant differences in scratch wound healing or transcription level compared to 1 h (* *p* < 0.05, ** *p* < 0.01, *** *p* < 0.001).

**Figure 2:**
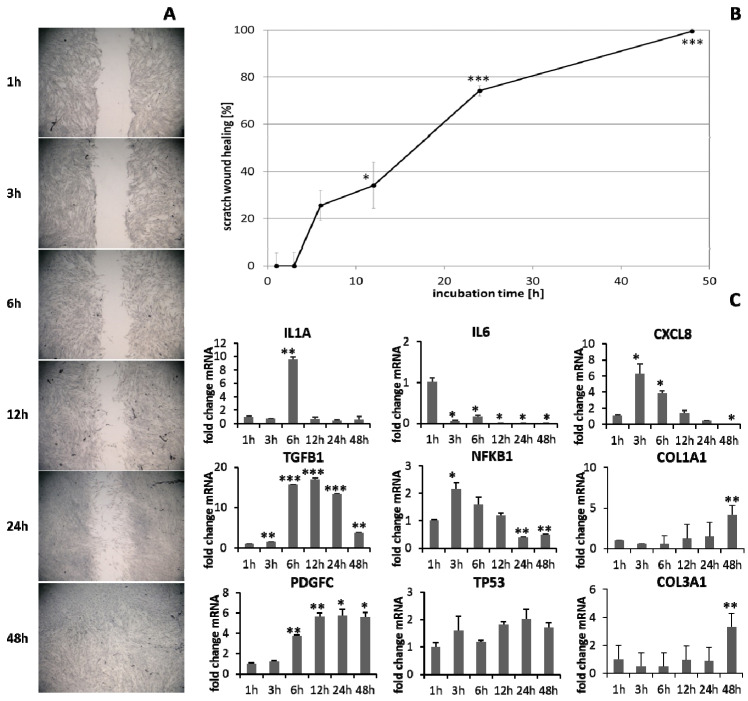
(**A**) Scratch wound healing of dermal fibroblasts under optimal conditions and (**B**) evaluation of scratch wound healing in [%]. (**C**) Gene expression profiles over time during wound healing. Asterisks [*] indicate significant differences in scratch wound healing or transcription level compared to 1 h (* *p* < 0.05, ** *p* < 0.01, *** *p* < 0.001).

HaCaT keratinocytes (Figure 1) and dermal fibroblasts (Figure 2) were investigated as model cells for epithelial and dermal wound healing. Scratch width declined over time; the first migrating cells could be noted at the time point after 12 h for keratinocytes (Figure 1A) and after 6 h for fibroblasts (Figure 2A). Wound healing progression, displayed as scratch wound healing in [%], reached almost 100% after 48 h for both cell types (*p* < 0.001; Figure 1B and Figure 2B). Furthermore, gene expression analysis was performed for pro-inflammatory cytokines, growth factors, transcription factors, and stress response genes as well as desmogleins such as cell adhesion molecules and antimicrobial peptides for keratinocytes along with collagen 1 and 3 in the case of fibroblasts to investigate their profiles during wound healing. It was found that with the progression of healing, the expression of pro-inflammatory cytokines gradually decreased in keratinocytes (Figure 1C) over time while a distinct pro-inflammatory reaction was observed in fibroblasts (Figure 2C) accompanied by an increase in *IL1A* expression most notably after 6 h (*p* < 0.01) and of *CXCL8* expression, reaching a peak as early as 3 h (*p* < 0.05). *TGFB1* expression increased over time in keratinocytes (Figure 1C) and showed a peak as early as 12 h in fibroblasts (*p* < 0.01; Figure 2C). A slight surge in *PDGFC* expression was observed for keratinocytes at 3 h with a subsequent decrease (Figure 1C) while fibroblasts demonstrated a steady rise in transcript levels over time under these conditions (*p* < 0.05; Figure 2C). Correspondingly, gene expression of *TP53* and *NFKB* was elevated during wound healing in keratinocytes (Figure 1C) and fibroblasts (Figure 2C). *DSG1* and *DSG3* expression by keratinocytes was significantly increased over time (*p* < 0.05; Figure 1C). Moreover, a significant increase in antimicrobial peptide gene expression by keratinocytes, such as *S100A7* (*p* < 0.01), *RNASE7* (*p* < 0.05), and *DEFB1* (*p* < 0.001) was noted with scratch wound healing (Figure 1B). It was also observed that, here, the fold changes in transcript levels were distinctly higher compared to those in the regulatory mediators. This was also observed for *DSG1*. Regeneration of the dermal layer was assessed by collagen expression in fibroblasts. *COL1A1* and *COL3A1* expression increased significantly over time corresponding to the reformation of the fibroblast cell layer (*p* < 0.01; Figure 2C).

### 3.2. Scratch Treatment with wIRA and UV-B Radiation

**Figure 3:**
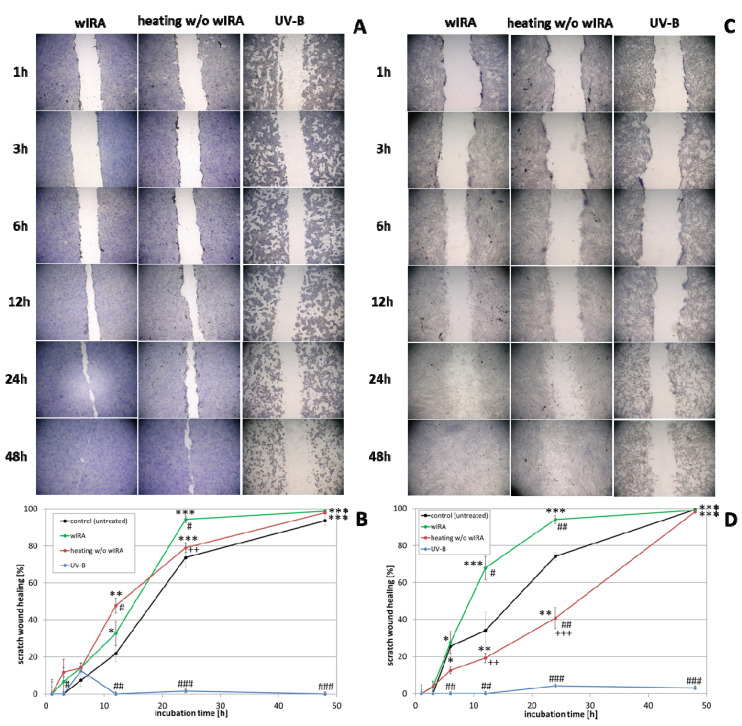
(**A**) HaCaT keratinocyte scratch wound closure over time after wIRA radiation, heating *w*/*o* wIRA and UV-B irradiation and (**B**) evaluation of scratch wound healing in [%]. (**C**) Closure of dermal fibroblast scratches over 48 h after wIRA radiation, heating *w*/*o* wIRA and UV-B irradiation, and (**D**) the evaluation of scratch wound healing progression in [%]. Asterisks [*] indicate significant differences in scratch wound healing or transcription level compared to 1 h (* *p* < 0.05, ** *p* < 0.01, *** *p* < 0.001). Hashtags [#] designate significant differences of scratch wound healing or transcription levels at the respective time point compared to the control under optimal conditions (# *p* < 0.05, ## *p* < 0.01, ### *p* < 0.001) while the plus sign [+] specifies significant differences between wIRA-treated samples and those receiving heating *w*/*o* wIRA under non-optimal conditions at the respective time point (++ *p* < 0.01, +++ *p* < 0.001).

The effect of wIRA radiation, heating *w*/*o* wIRA (as thermal control) and UV-B irradiation on scratch wound healing of keratinocytes (Figure 3A,B) and fibroblasts (Figure 3C,D) was investigated under optimal conditions for the cells, referring to a physiological cell environment. Keratinocyte scratches showed an improvement in wound healing after treatment with wIRA (Figure 3A,B) with a significantly increased scratch closure at 24 h (*p* < 0.01). Moreover, wIRA radiation distinctly improved wound healing of fibroblast scratches in vitro (Figure 3C,D) with a significantly increased scratch closure at 12 h (*p* < 0.05). Heating *w*/*o* wIRA for 20 min slightly improved keratinocyte scratch healing compared to the untreated control (*p* < 0.05), but delayed healing of fibroblast scratches (*p* < 0.01). UV-B irradiation resulted in severe damage of the keratinocytes (Figure 3A,B) and exerted a detrimental effect on fibroblasts (Figure 3C,D) resulting in scratch wounds that remained open and showed no tendency for healing (*p* < 0.001).

**Figure 4:**
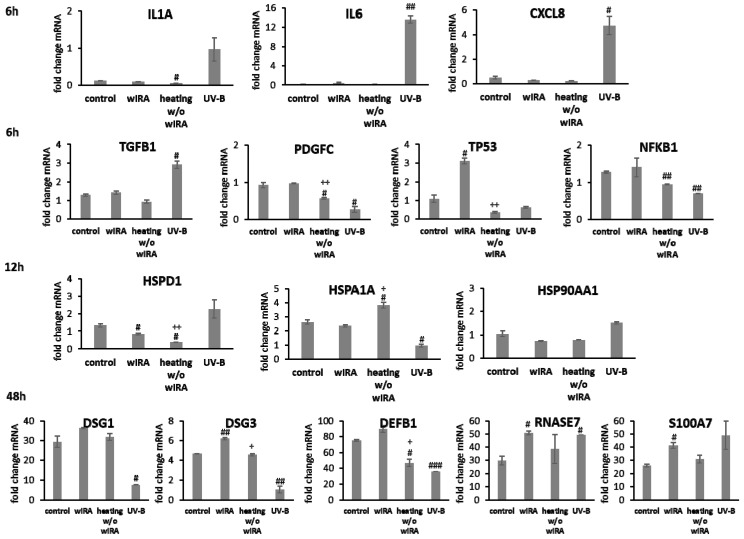
Gene expression profiles of pro-inflammatory cytokine genes and growth factor genes after 6 h and heat shock protein gene transcript levels after 12 h as well as desmoglein gene and AMP gene expression after 48 h for HaCaT keratinocyte scratches treated with wIRA, heating *w*/*o* wIRA and UV-B irradiation. Hashtags [#] designate significant differences of scratch wound healing or transcription levels at the respective time point compared to the control under optimal conditions (# *p* < 0.05, ## *p* < 0.01, ### *p* < 0.001) while the plus sign [+] specifies significant differences between wIRA-treated samples and those receiving heating *w*/*o* wIRA under non-optimal conditions at the respective time point (+ *p* < 0.05, ++ *p* < 0.01).

Transcript levels of *IL1A*, *IL6* (*p* < 0.01) and *CXCL8* (*p* < 0.05) were distinctly elevated at 6 h after UV-B irradiation in keratinocytes possibly resulting in the detrimental activation of the inflammatory response (Figure 4). Radiation with wIRA did not change pro-inflammatory cytokine expression compared to the untreated control. A significant decrease of *IL1A* was noted at 6 h after heating *w*/*o* wIRA (*p* < 0.05). Physiological levels of *TGFB1* were noted after treatment with wIRA but decreased gene expression was found after heating alone. Surprisingly, UV-B exposure significantly induced *TGFB1* expression at 6 h after irradiation (*p* < 0.05; Figure 4). *PDGFC* expression showed no distinct changes with wIRA, but was markedly reduced at 6 h after heating *w*/*o* wIRA (*P* < 0.05) and UV-B irradiation (*p* < 0.05), as were the *NFKB1* levels (*p* < 0.01) and *TP53* gene expression (Figure 4). In contrast, *TP53* levels were significantly elevated 6 h after wIRA radiation compared to the control (*p* < 0.05). Heat shock proteins such as HSP90, HSPA1, and HSPD1 possess crucial roles as chaperones in protein folding and as possible signaling regulators inducing cellular stress responses [65,66,67]. In accordance, induction of *HSP90AA1*, *HSPA1A*, and *HSPD1* gene expression was evaluated after the use of light and heat emitting devices. Stress response genes were differently transcribed in wIRA-treated and UV-B-irradiated keratinocytes after scratching at 12 h (Figure 4). *HSPA1A* was readily increased by heating *w*/*o* wIRA compared to the control (*p* < 0.05) and significantly decreased by UV-B irradiation (*p* < 0.05). However, expression of this gene was not altered after wIRA treatment. *HSP90AA1* and *HSPD1* transcription was slightly increased by UV-B irradiation without reaching statistical significance, while wIRA (*p* < 0.05), and especially heating alone, markedly decreased *HSPD1* levels at 12 h (*p* < 0.05). Transcript levels of the cell adhesion molecule *DSG1* were slightly higher and gene expression of *DSG3* significantly elevated (*p* < 0.01) after wIRA treatment compared to the control (Figure 4), possibly corresponding to the differences observed in the healing progression over time (Figure 3B). Significantly reduced amounts of *DSG1* and *DSG3* were observed after UV-B irradiation (*p* < 0.05). The antimicrobial peptide genes were differently transcribed after 48 h dependent on the treatment (Figure 4). *DEFB1* levels were elevated by wIRA while heating alone (*p* < 0.05) and UV-B irradiation (*p* < 0.01) significantly reduced it. *RNASE7* and *S100A7* were increased by wIRA (*p* < 0.05) and UV-B compared to the control while heating alone had a lesser effect.

**Figure 5:**
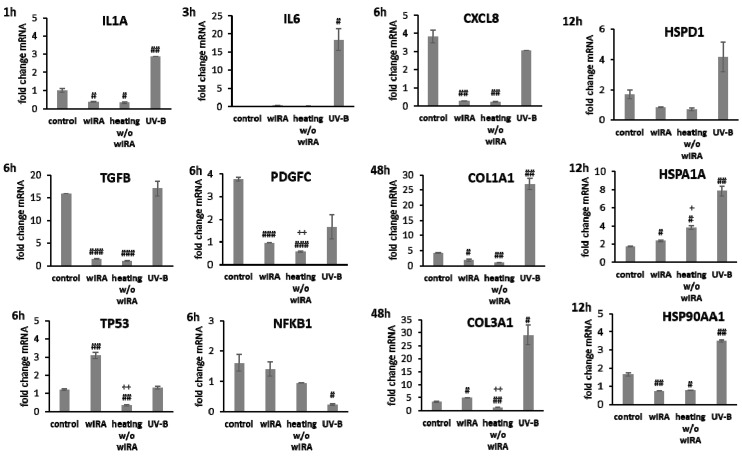
Gene expression profiles of pro-inflammatory cytokine genes after 1 h (*IL1A*), 3 h (*IL6*), and 6 h (*CXCL8*) for dermal fibroblasts scratches treated with wIRA, heating *w*/*o* wIRA and UV-B irradiation. Furthermore, differences for growth factor gene expression were evaluated after 6 h and heat shock protein gene transcript levels after 12 h as well as collagen gene transcription after 48 h. Hashtags [#] designate significant differences of scratch wound healing or transcription levels at the respective time point compared to the control under optimal conditions (# *p* < 0.05, ## *p* < 0.01, ### *p* < 0.001) while the plus sign [+] specifies significant differences between wIRA-treated samples and those receiving heating *w*/*o* wIRA under non-optimal conditions at the respective time point (+ *p* < 0.05, ++ *p* < 0.01).

In fibroblasts irradiated with UV-B, *IL1A* expression after 1 h, *IL6* levels after 3 h, and *CXCL8* amounts after 6 h were distinctly elevated compared to wIRA and heating alone (Figure 5). After wIRA treatment and heating alone, *IL1A* (*p* < 0.05) and *CXCL8* (*p* < 0.01) were significantly reduced compared to the control. *TGFB1* expression at 6 h after UV-B irradiation was comparable to the control but without eliciting cell migration or proliferation and distinctly higher fold changes in mRNA levels in fibroblasts compared to keratinocytes were noted. *PDGFC* was distinctly reduced compared to the control (Figure 5). *TGFB1* and *PDGFC* gene expression were also significantly reduced by wIRA treatment and heating *w*/*o* wIRA at 6 h (*p* < 0.001), which would match the absent inflammatory response. However, growth factor gene expression increased at later time points, where it corresponded with the healing progression. *TP53* transcription was significantly induced in fibroblasts by wIRA radiation after 6 h (*p* < 0.01) but not by heating alone (*p* < 0.01). *NFKB* gene expression by fibroblasts after treatment with wIRA corresponded to the untreated control while heating *w*/*o* wIRA slightly and UV-B irradiation significantly (*p* < 0.05) reduced *NFKB* transcript levels in fibroblasts (Figure 5). Stress response gene transcription analysis revealed a distinct induction of *HSP90AA1* and *HSPA1A* expression in fibroblasts by UV-B irradiation after 12 h (*p* < 0.01) comparable to keratinocytes together with an increase in *HSPD1* levels (Figure 5). *HSPA1A* was also readily induced by heating *w*/*o* wIRA (*p* < 0.05), but affected by wIRA treatment to a much lesser extent (*p* < 0.05) while *HSP90AA1* (*p* < 0.01) and *HSPD1* levels in fibroblasts were reduced by wIRA radiation at this time point (Figure 5). *COL3A1* was found to be significantly increased after wIRA treatment (*p* < 0.01) compared to heating *w*/*o* wIRA (Figure 5), corresponding to the healing progression observed (Figure 3D). Interestingly, a most pronounced increase in *COL1A1* (*p* < 0.01) and *COL3A1* (*p*< 0.05) was also observed after UV-B irradiation in the fibroblasts.

### 3.3. Scratch Wound Healing under Non-Optimal Conditions

**Figure 6:**
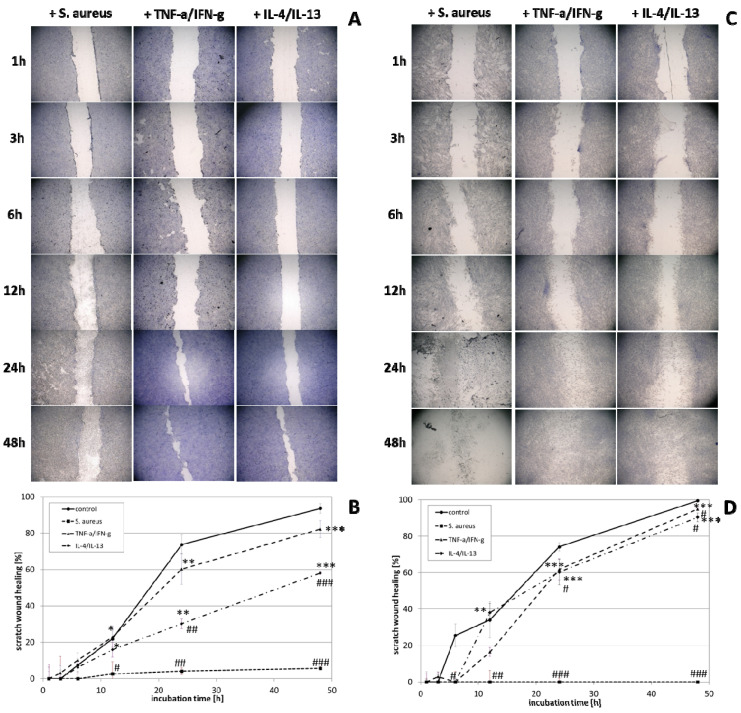
(**A**) HaCaT keratinocyte scratch wound closure over time after infection with *S. aureus* and induction of chronic inflammatory conditions (TNF-α/IFN-γ) as well as anti-inflammatory conditions (IL-4/IL-13) and (**B**) evaluation of scratch wound healing in [%]. (**C**) Closure of dermal fibroblast scratches over 48 h after infection with *S. aureus* and induction of chronic inflammatory conditions (TNF-α/IFN-γ) as well as anti-inflammatory conditions (IL-4/IL-13) and (**D**) the evaluation of scratch wound healing progression in [%]. Asterisks [*] indicate significant differences in scratch wound healing or transcription level compared to 1 h (* *p* < 0.05, ** *p* < 0.01, *** *p* < 0.001). Hashtags [#] designate significant differences of scratch wound healing or transcription levels at the respective time point compared to the control under optimal conditions (# *p* < 0.05, ## *p* < 0.01, ### *p* < 0.001).

The experimental prerequisites in scratch assays mostly account for optimal cell conditions. Hence, the next step of the study included the readjustment of specific milieus more closely mimicking different in vivo situations, explicitly *S. aureus* infection, chronic inflammation, and anti-inflammatory conditions. Scratch wound healing progression in keratinocytes (Figure 6A,B) and fibroblasts (Figure 6C,D) was distinctly reduced under all non-optimal conditions. The influence was most pronounced for the infection with *S. aureus (p* < 0.001), which damaged the cells to an extent that scratch wounds could not be closed neither by keratinocytes nor fibroblasts. *S. aureus* overgrowth was noted after 12 h, most likely inducing cell death. In accordance, no living cells were noted 24 and 48 h after infection and, consequently, mRNA gene expression analysis could not be performed at these time points. Under anti-inflammatory conditions, where cells were primed with IL-4/IL-13 for 24 h prior to scratching, keratinocyte scratch closure was also significantly impeded, reaching only 60% scratch healing after 48 h (*p* < 0.001; Figure 6B). The scratch wound healing with fibroblasts was also slightly impeded, achieving only 90% closure after 48 h (*p* < 0.05; Figure 6D). Chronic inflammatory conditions generated by priming with TNF-α/IFN-γ reduced scratch healing compared to the keratinocyte control under optimal conditions (Figure 6B). The effect of the chronic inflammatory setting was even more pronounced on fibroblasts, reducing cell proliferation and migration at early time points (*p* < 0.05; Figure 6D).

**Figure 7:**
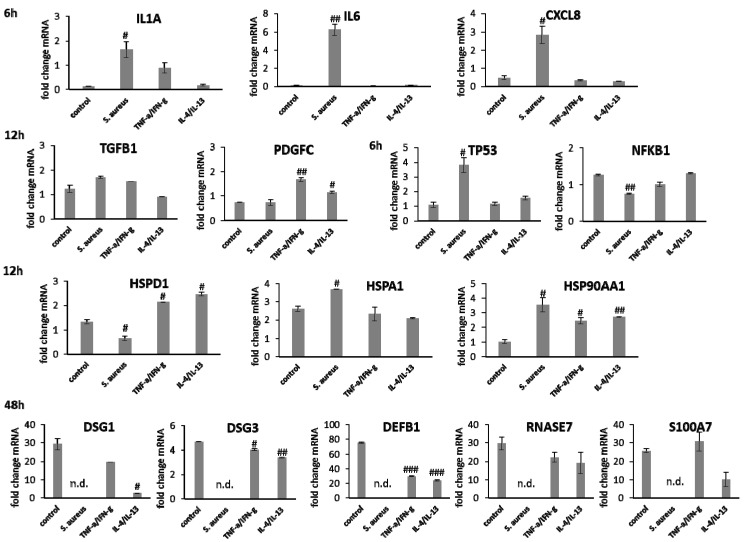
Gene expression profiles of pro-inflammatory cytokine and growth factor genes after 6 h and heat shock protein gene transcript levels after 12 h as well as desmoglein gene and AMP gene expression after 48 h for HaCaT keratinocyte scratches after infection with *S. aureus* and induction of chronic inflammatory conditions (TNF-α/IFN-γ) as well as anti-inflammatory conditions (IL-4/IL-13). Hashtags [#] designate significant differences of scratch wound healing or transcription levels at the respective time point compared to the control under optimal conditions (# *p* < 0.05, ## *p* < 0.01, ### *p* < 0.001) and n.d. indicates ‘not determined’.

**Figure 8:**
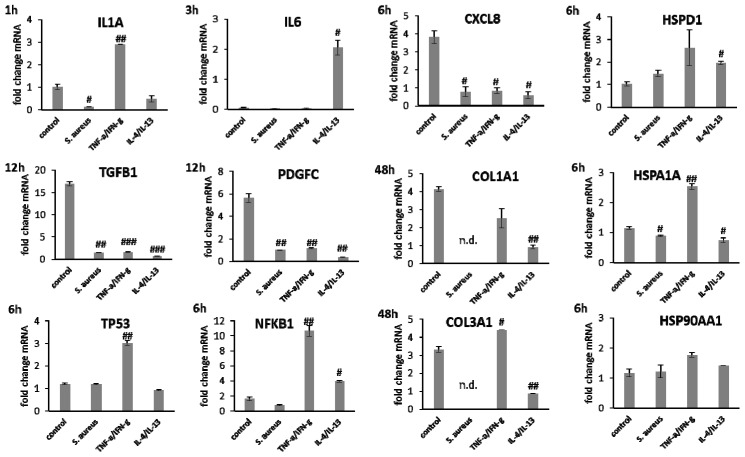
Gene expression profiles of pro-inflammatory cytokine genes after 1 h (*IL1A*), 3 h (*IL6*), and 6 h (*CXCL8*) for fibroblast scratches after infection with *S. aureus* and induction of chronic inflammatory conditions (TNF-α/IFN-γ) as well as anti-inflammatory conditions (IL-4/IL-13). Stress protein response was further evaluated after 6 h at the level of *TP53* and *NFKB1* transcripts as well as heat shock protein and growth factor gene expression were assessed after 12 h. Differences in collagen gene expression were determined after 48 h. Hashtags [#] designate significant differences of scratch wound healing or transcription levels at the respective time point compared to the control under optimal conditions (# *p* < 0.05, ## *p* < 0.01, ### *p* < 0.001) and n.d. indicates ‘not determined’.

Gene expression analysis revealed a significant induction in pro-inflammatory cytokine transcription in keratinocytes during infection with *S. aureus* with induction of *IL1A (p* < 0.05), *IL6* (*p* < 0.01), and *CXCL8* (*p* < 0.05) at early time points (Figure 7). In contrast, infection with *S. aureus* failed to elicit pro-inflammatory cytokine gene expression in fibroblasts, most likely due to the associated toxic effects of *S. aureus* (Figure 8). A pro-inflammatory milieu led to slightly increased *IL1A* mRNA levels in keratinocytes at 6 h compared to the control (Figure 7). *IL6* and *CXCL8* expression showed no differences to the control (Figure 7). Accordingly, *IL1A* transcription by fibroblasts was markedly increased (*p* < 0.01) at one hour under chronic inflammatory conditions (Figure 8). Surprisingly, *IL-6* was induced in IL-4/IL-13-stimulated fibroblasts 3 h after scratching (*p* < 0.05) while *CXCL8* was elevated at 6 h under optimal conditions (Figure 8), demonstrating a significant difference to all non-optimal conditions (*p* < 0.05). *PDGFC* levels significantly increased under chronic inflammatory conditions (*p* < 0.01) while *TGFB1* was only slightly enhanced (Figure 7), but corresponded to the keratinocyte scratch wound healing progression (Figure 6B). Though, *TGFB1* and *PDGFC* also increased under chronic inflammatory conditions in fibroblasts over time, the response was markedly decreased compared to optimal conditions (*p* < 0.01; Figure 8), reflecting the slower healing progression (Figure 6D). No noteworthy *TGFB1* upregulation was observed under anti-inflammatory conditions in both cell types (Figure 7 and Figure 8), probably accounting for the reduced scratch healing observed (Figure 6B,D). In contrast, *PDGFC* was significantly increased (*p* < 0.05) at 12 h in keratinocytes (Figure 7), possibly being the result of a delayed response. Moreover, *TP53* was increased in infected keratinocytes (*p* < 0.05) while *NFKB1* was reduced (*p* < 0.01) at 6 h (Figure 7). In contrast, *S. aureus* infection did not change transcription factor transcript levels in fibroblast scratches after 6 h (Figure 8). Anti-inflammatory conditions also slightly induced *TP53* in scratched keratinocytes at 6 h (Figure 7) and significantly increased *NFKB1* levels in fibroblasts (*p* < 0.05; Figure 8). While the gene expression of transcription factors was not affected by chronic inflammatory conditions in keratinocytes compared to the control under optimal conditions (Figure 7), it was significantly increased in fibroblasts at 6 h (*p* < 0.01; Figure 8). Heat shock protein gene expression evaluation pointed to condition-specific increases in *HSP90AA1*, *HSPA1A*, and *HSPD1* transcript levels under the non-optimal conditions compared to the keratinocyte control (Figure 7). Chronic and anti-inflammatory conditions elicited an increase in *HSPD1* levels (*p* < 0.05) while *S. aureus* reduced its transcription in keratinocytes (*p* < 0.05). *HSPA1A* and *HSP90AA1* were induced by *S. aureus (p* < 0.05). *HSP90AA1* transcription was also significantly stimulated by pro- and anti-inflammatory conditions compared to the control. Non-optimal conditions in fibroblasts resulted in increased stress response gene transcription with marked effects on *HSPD1* for TNF-α/IFN-γ stimulation, which were also found for *HSPA1A (p* < 0.01) and to a lesser extent *HSP90AA1* (Figure 8). Minor effects on *HSPD1* were found for infection with *S. aureus*, which did not reach statistical significance, and scratch wound healing under anti-inflammatory conditions (*p* < 0.05). *DSG1* levels under chronic inflammatory conditions were slightly decreased compared to the keratinocyte control and *DSG3* was significantly affected (Figure 7), which possibly reflects the reduced scratch wound healing progression (Figure 6B). A markedly reduced transcription of these cell adhesion molecule genes was found under anti-inflammatory conditions (*p* < 0.05), also corresponding to the decreased wound healing under these conditions in vitro. Gene expression of the antimicrobial peptides *S100A7* and *RNASE7* was induced by *S. aureus* infection at early time points but could not be determined at 48 h due to the loss of viable cells (Figure 7). In contrast, *S100A7* was not changed under chronic inflammatory conditions, while *RNASE7* transcription was slightly decreased and *DEFB1* levels were significantly reduced (*p* < 0.001) in keratinocytes. Anti-inflammatory conditions generally reduced antimicrobial peptide gene expression, reaching high statistical significance for *DEFB1* (*p* < 0.001). *COL1A1* and *COL3A1* transcription by fibroblasts increased with the healing progression over time under chronic inflammatory conditions, but while *COL3A1* even exceeded the control levels (*p* < 0.05), *COL1A1* was reduced compared to the optimal conditions. After infection with *S. aureus*, no determination of collagen gene expression was possible after 48 h due to the loss of viable cells. Anti-inflammatory conditioning with IL-4/IL-13 significantly reduced *COL1A1* and *COL3A1* transcription (*p* < 0.01; Figure 8).

### 3.4. wIRA Treatment of Cell Scratches during S. aureus Infection

**Figure 9:**
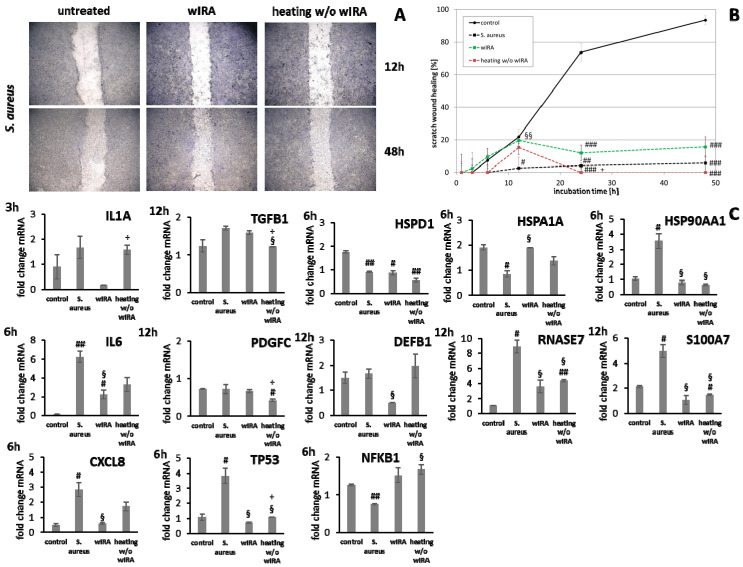
(**A**) HaCaT keratinocyte scratch wound closure over time after infection with *S. aureus* and treatment with wIRA or heating *w*/*o* wIRA. (**B**) Evaluation of scratch wound healing in [%]. (**C**) Gene expression profiles of these HaCaT keratinocytes for pro-inflammatory cytokine genes after 3 h (*IL1A*) and 6 h (*IL6*, *CXCL8*) together with stress response gene and heat shock protein gene transcript levels as well as growth factor and AMP gene expression after 12 h. Hashtags [#] designate significant differences of scratch wound healing or transcription levels at the respective time point compared to the control under optimal conditions (# *p* < 0.05, ## *p* < 0.01, ### *p* < 0.001) while the paragraph character [§] shows significant differences between untreated and treated samples under non-optimal conditions at the respective time point (§ *p* < 0.05, §§ *p* < 0.001). The plus sign [+] specifies significant differences between wIRA-treated samples and those receiving heating *w*/*o* wIRA under non-optimal conditions at the respective time point (+ *p* < 0.05).

**Figure 10:**
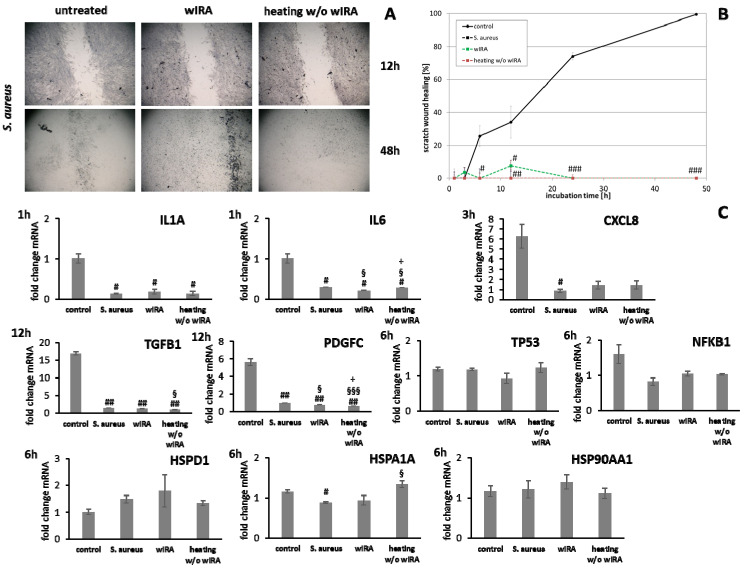
(**A**) Dermal fibroblast scratch wound closure over time after infection with *S. aureus* and treatment with wIRA or heating *w*/*o* wIRA. (**B**) Evaluation of scratch wound healing in [%]. (**C**) Gene expression profiles of these fibroblast scratches for pro-inflammatory cytokine genes after 1 h (*IL1A*, *IL6*) and 3 h (*CXCL8*) as well as stress response gene and heat shock protein transcript levels. Growth factor gene expression was evaluated after 12 h. Hashtags [#] designate significant differences of scratch wound healing or transcription levels at the respective time point compared to the control under optimal conditions (# *p* < 0.05, ## *p* < 0.01, ### *p* < 0.001) while the paragraph character [§] shows significant differences between untreated and treated samples under non-optimal conditions at the respective time point (§ *p* < 0.05, §§§ *p* < 0.001). The plus sign [+] specifies significant differences between wIRA-treated samples and those receiving heating *w*/*o* wIRA under non-optimal conditions at the respective time point (+ *p* < 0.05).

Keratinocyte and fibroblast monolayers were infected with *S. aureus* and scratched before treatment with wIRA or heating *w*/*o* wIRA. In all cases, infection with *S. aureus* damaged the cells so severely that scratch wound closure was inhibited independent of the treatment (Figure 9A,B and Figure 10A,B). Moreover, no living cells were noted after 24 and 48 h under infection conditions in the untreated control and after heating *w*/*o* wIRA. Treatment with wIRA slightly stalled the negative outcome in keratinocytes (*p* < 0.01) and those remained viable up to 24 h but eventually succumbed to the toxic effects after 48 h (Figure 9A). Radiation with wIRA showed no protective effect on fibroblasts (Figure 10A). Consequently, at the time points where cells were killed, no mRNA gene expression analysis was performed.

Treatment with wIRA decreased *IL1A* levels at 3 h as well as *IL6* (*p* < 0.05) and *CXCL8* (*p* < 0.05) transcription after 6 h in infected keratinocytes while heating alone showed no or only slight effects (Figure 9C). In fibroblasts, the inflammatory response after *S. aureus* infection was decreased and wIRA treatment as well as heating *w*/*o* wIRA did not markedly change *IL1A* and *CXCL8* transcription profiles at early time points (Figure 10C), only *IL6* levels were significantly decreased (*p* < 0.05). *TGFB1* and *PDGFC* levels in infected keratinocytes were neither affected by wIRA treatment compared to the infected cells while heating *w*/*o* wIRA further decreased their levels (*p* < 0.05). Similar results were obtained for fibroblasts (Figure 10C). Transcription of *TP53* by keratinocytes was increased at 6 h under infection conditions, which was found to be significantly reduced in wIRA-treated keratinocytes and for those treated by heating *w*/*o* wIRA (*p* < 0.05). *NFKB1* was augmented with wIRA treatment and especially heating alone (*p* < 0.05) compared to infected cells (Figure 9C). Moreover, it was found that wIRA radiation, and to some extent, heating *w*/*o* wIRA, could restore *HSPA1A* transcription to control levels while *HSPD1* expression was not affected (Figure 9C). In contrast, the *HSP90AA1* increase in infected keratinocytes was significantly alleviated at 6 h under wIRA treatment and heating of the cells (*p* < 0.05; Figure 9C). Changes in heat shock protein transcript levels after infection of fibroblasts with *S. aureus* and treatment with wIRA were observed for *HSPD1* as a slight increase and with heating *w*/*o* wIRA for *HSPA1A* as significant enhancement (*p* < 0.05) compared to infected cells (Figure 10C). Transcription of the antimicrobial peptides *S100A7* and *RNASE7*, but not *DEFB1* was distinctly induced by *S. aureus* infection in keratinocytes (*p* < 0.05) compared to the control at 12 h (Figure 9C). Heating *w*/*o* wIRA did not affect *DEFB1* expression, but significantly reduced *S100A7* and *RNASE7* expression (*p* < 0.05). Surprisingly, the wIRA treatment led to overall decreased AMP gene transcript levels in the infected keratinocytes (*p* < 0.05; Figure 9C).

### 3.5. Stimulation of Scratch Wound Healing under Chronic Inflammatory Conditions by wIRA

**Figure 11:**
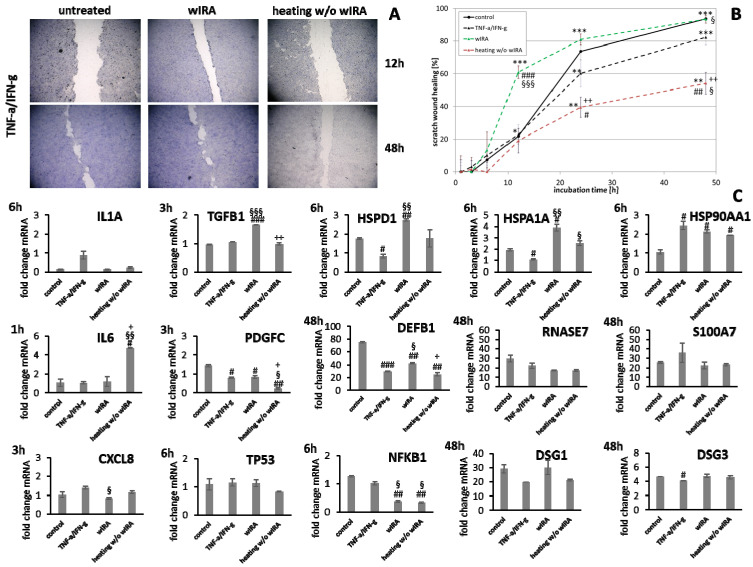
(**A**) HaCaT keratinocyte scratch wound closure over time after induction of chronic inflammatory conditions (TNF-α/IFN-γ) and treatment with wIRA or heating *w*/*o* wIRA. (**B**) Evaluation of scratch wound healing in [%]. (**C**) Gene expression profiles of these HaCaT keratinocytes at specific time points during scratch wound healing after induction of chronic inflammatory conditions (TNF-α/IFN-γ) showed differences for pro-inflammatory cytokine gene expression after 1 h (*IL1A*, *IL6*) and 3 h (*CXCL8*) as well as growth factor gene transcription after 3 h and stress response gene levels after 6 h. In addition, heat shock protein gene expression exhibited alterations after 6 h while AMP gene expression was partly affected at 48 h as well as desmoglein gene expression. Asterisks [*] indicate significant differences in scratch wound healing or transcription level compared to 1 h (* *p* < 0.05, ** *p* < 0.01, *** *p* < 0.001). Hashtags [#] designate significant differences of scratch wound healing or transcription levels at the respective time point compared to the control under optimal conditions (# *p* < 0.05, ## *p* < 0.01, ### *p* < 0.001) while the paragraph character [§] shows significant differences between untreated and treated samples under non-optimal conditions at the respective time point (§ *p* < 0.05, §§ *p* < 0.001, §§§ *p* < 0.001). The plus sign [+] specifies significant differences between wIRA-treated samples and those receiving heating *w*/*o* wIRA under non-optimal conditions at the respective time point (+ *p* < 0.05, ++ *p* < 0.01).

**Figure 12:**
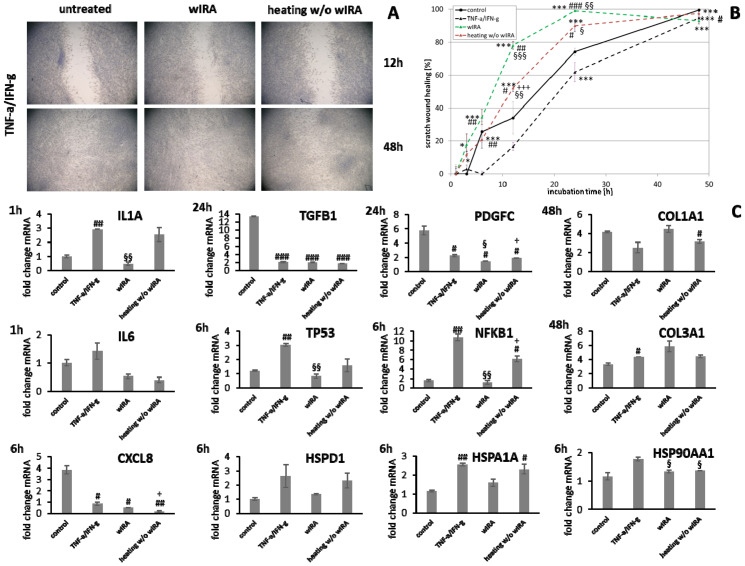
(**A**) Dermal fibroblast scratch wound closure over time after induction of chronic inflammatory conditions (TNF-α/IFN-γ) and treatment with wIRA or heating *w*/*o* wIRA. (**B**) Evaluation of scratch wound healing in [%]. (**C**) Gene expression profiles of these fibroblasts were assessed for pro-inflammatory cytokine gene expression after 1 h (*IL1A*, *IL6*) and 6 h (*CXCL8*) as well as stress response gene together with heat shock protein gene transcript levels. Growth factor gene expression was appraised after 24 h and collagen gene expression was evaluated after 48 h. Asterisks [*] indicate significant differences in scratch wound healing or transcription level compared to 1 h (* *p* < 0.05, *** *p* < 0.001). Hashtags [#] designate significant differences of scratch wound healing or transcription levels at the respective time point compared to the control under optimal conditions (# *p* < 0.05, ## *p* < 0.01, ### *p* < 0.001) while the paragraph character [§] shows significant differences between untreated and treated samples under non-optimal conditions at the respective time point (§ *p* < 0.05, §§ *p* < 0.001, §§§ *p* < 0.001). The plus sign [+] specifies significant differences between wIRA-treated samples and those receiving heating *w*/*o* wIRA under non-optimal conditions at the respective time point (+ *p* < 0.05, +++ *p* < 0.01).

Keratinocytes were primed with TNF-α/INF-γ to induce a state of chronic inflammation. These monolayers were scratched and treated with wIRA or heating *w*/*o* wIRA (Figure 11A,B). Heating *w*/*o* wIRA exerted a negative effect on the keratinocytes and scratch wound healing was significantly decreased (*p* < 0.01). In contrast, heating alone was enough to significantly improve fibroblast scratch wound closure as early as 12 h (*p* < 0.01) compared to the untreated control under chronic inflammatory conditions (Figure 12A,B). Treatment with wIRA significantly enhanced the wound healing capacity of keratinocytes under chronic inflammatory conditions (Figure 11A,B) with an increased scratch closure at 48 h (*p* < 0.05) and also induced the wound healing progression rate in fibroblast scratches as early as 12 h (*p* < 0.01) beyond the extent of cells under optimal conditions (Figure 12A,B).

Under chronic inflammatory conditions, an increased expression of *IL1A* was noted, which could be decreased by wIRA radiation in both keratinocytes (Figure 11C) and also significantly in fibroblasts (*p* < 0.01; Figure 12C). Heating *w*/*o* wIRA also decreased *IL1A* levels in keratinocytes (Figure 11C), but not in fibroblasts (Figure 12C) while it reduced *IL6* levels in the latter (Figure 12C) and significantly stimulated this cytokine’s gene transcription in keratinocytes (Figure 11C). *CXCL8* was significantly reduced by wIRA treatment under chronic inflammatory conditions in keratinocytes (*p* < 0.05) and slightly decreased in fibroblasts. *TGFB1* transcription in keratinocytes was significantly enhanced by wIRA (*p* < 0.001) while it remained unchanged between treatments in fibroblasts and here did not correspond with the overall scratch wound healing progression (Figure 12B). *PDGFC* transcription in keratinocytes was not altered under chronic inflammatory conditions by wIRA treatment, but it was found to be significantly reduced after heating *w*/*o* wIRA (*p* < 0.05; Figure 11C). No differences in *TP53* levels between treated and untreated keratinocyte scratches during wound healing progression were observed but *NFKB1* was significantly reduced in treated keratinocytes at 6 h (*p* < 0.01; Figure 11C). In addition, wIRA radiation significantly decreased *TP53* and *NFKB1* levels in fibroblasts (*p* < 0.01; Figure 12C). Keratinocytes transcribed increased levels of the heat shock protein gene *HSP90AA1* under chronic inflammatory conditions, which was not affected by treatments. However, *HSPD1* and *HSPA1A* were found to be significantly augmented by wIRA radiation under TNF-α/IFN-γ conditions (*p* < 0.01) as well as compared to the control (Figure 11C). In contrast, all heat shock proteins *HSP90AA1*, *HSPA1A*, and *HSPD1* were induced in fibroblasts by chronic inflammatory conditions. Their transcription was markedly decreased in wIRA-treated compared to untreated fibroblast scratches or those treated by heating *w*/*o* wIRA with the exception of *HSP90AA1*, which was also reduced by heating alone (Figure 12C). *DSG1* and *DSG3* levels under chronic inflammatory conditions after all treatments reflected scratch wound healing progression in keratinocytes. Transcription levels of the antimicrobial peptides *S100A7* and *RNASE7* were mostly unchanged by treatments compared to the untreated control under chronic inflammatory conditions. In contrast, *DEFB1* was significantly increased after wIRA radiation (*p* < 0.05), but did not reach the control levels of keratinocytes under optimal conditions during wound healing progression (Figure 11C). Treatment with wIRA enhanced *COL1A1* and *COL3A1* levels in fibroblasts compared to untreated scratches under chronic inflammatory conditions (Figure 12C), but changes did not reach statistical significance, reflecting the improved scratch wound healing progression (Figure 12B).

### 3.6. wIRA Treatment Accelerates Scratch Wound Healing under Anti-Inflammatory Conditions

**Figure 13:**
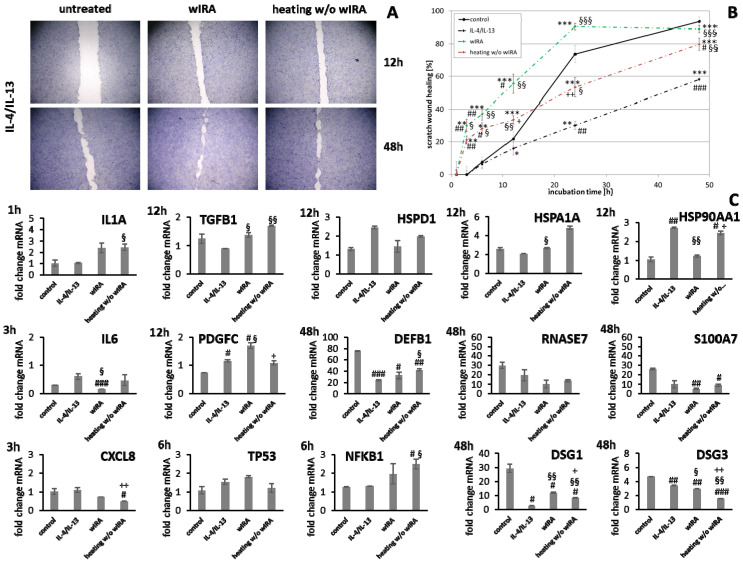
(**A**) HaCaT keratinocyte scratch wound closure over time after induction of anti-inflammatory conditions (IL-4/IL-13) and treatment with wIRA or heating *w*/*o* wIRA. (**B**) Evaluation of scratch wound healing in [%]. (**C**) Gene expression profiles of these HaCaT keratinocytes at specific time points during scratch wound healing after the induction of anti-inflammatory conditions demonstrated differences for pro-inflammatory cytokine gene expression after 1 h (*IL1A*) and 3 h (*IL6*, *CXCL8*) as well as stress response gene transcript levels after 6 h and growth factor gene transcript levels after 12 h. Additionally, heat shock protein gene expression and AMP gene expression exhibited alterations at 48 h as well as desmoglein gene expression. Asterisks [*] indicate significant differences in scratch wound healing or transcription level compared to 1 h (* *p* < 0.05, ** *p* < 0.01, *** *p* < 0.001). Hashtags [#] designate significant differences of scratch wound healing or transcription levels at the respective time point compared to the control under optimal conditions (# *p* < 0.05, ## *p* < 0.01, ### *p* < 0.001) while the paragraph character [§] shows significant differences between untreated and treated samples under non-optimal conditions at the respective time point (§ *p* < 0.05, §§ *p* < 0.001, §§§ *p* < 0.001). The plus sign [+] specifies significant differences between wIRA-treated samples and those receiving heating *w*/*o* wIRA under non-optimal conditions at the respective time point (+ *p* < 0.05, ++ *p* < 0.01).

**Figure 14:**
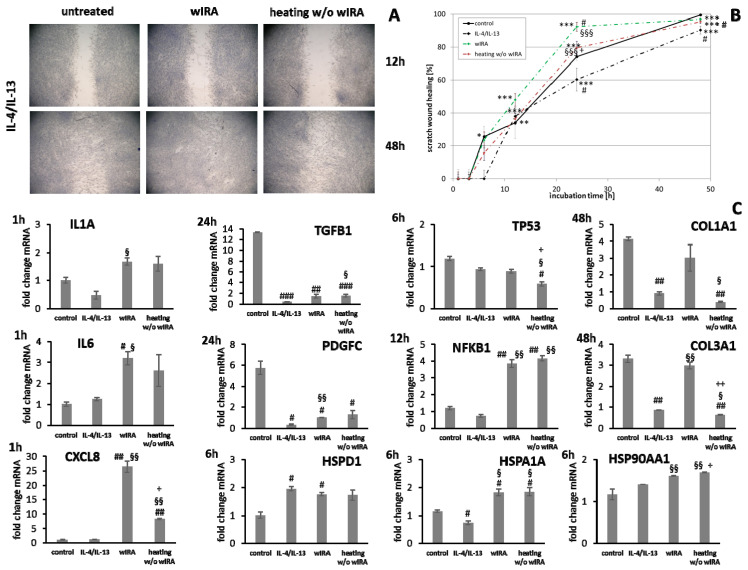
(**A**) Dermal fibroblast scratch wound closure over time after induction of anti-inflammatory conditions (IL-4/IL-13) and treatment with wIRA or heating *w*/*o* wIRA. (**B**) Evaluation of scratch wound healing in [%]. (**C**) Gene expression profiles of these fibroblasts were appraised for pro-inflammatory cytokine gene expression after 1 h and heat shock protein gene transcript levels after 6 h. Furthermore, stress response gene expression exhibited differences after 6 h (*TP53*) and 12 h (*NFKB1*). Growth factor gene expression was assessed after 24 h and collagen gene expression was evaluated after 48 h. Asterisks [*] indicate significant differences in scratch wound healing or transcription level compared to 1 h (* *p* < 0.05, ** *p* < 0.01, *** *p* < 0.001). Hashtags [#] designate significant differences of scratch wound healing or transcription levels at the respective time point compared to the control under optimal conditions (# *p* < 0.05, ## *p* < 0.01, ### *p* < 0.001) while the paragraph character [§] shows significant differences between untreated and treated samples under non-optimal conditions at the respective time point (§ *p* < 0.05, §§ *p* < 0.001, §§§ *p* < 0.001). The plus sign [+] specifies significant differences between wIRA-treated samples and those receiving heating *w*/*o* wIRA under non-optimal conditions at the respective time point (+ *p* < 0.05, ++ *p* < 0.01).

Scratch wound healing was found to be distinctly reduced by the addition of IL-4/IL-13, mimicking anti-inflammatory conditions. Heating *w*/*o* wIRA increased overall keratinocyte scratch healing (Figure 13A,B, *p* < 0.01) and induced early scratch healing progression in fibroblasts (Figure 14A,B, *p* < 0.001) under anti-inflammatory conditions. In contrast, wIRA radiation highly significantly enhanced scratch wound healing in keratinocytes (*p* < 0.001) and fibroblasts (*p* < 0.001) beyond the extent of cells under optimal conditions.

*IL1A* gene expression by keratinocytes after treatments was distinctly elevated at early time points compared to the untreated control (Figure 13C). *IL6* transcription under anti-inflammatory conditions was significantly reduced by wIRA (*p* < 0.05) and *CXCL8* levels were not or only marginally affected by the treatments (Figure 13C). In fibroblasts, *IL1A* levels were increased by both treatments under anti-inflammatory conditions at one hour while *IL6* (*p* < 0.05) and *CXCL8* (*p* < 0.01) were distinctly induced after wIRA radiation and to a lesser extent by heating *w*/*o* wIRA (Figure 14C). Particularly noticeable was the significant increase in mRNA level fold change for *CXCL8*. A significant *TGFB1* upregulation was observed in keratinocytes after treatments compared to the cells under anti-inflammatory conditions alone (*p* < 0.05) and *PDGFC* expression was significantly increased by wIRA radiation at 12 h (*p* < 0.05; Figure 13C). *TGFB1* and *PDGFC* transcription were also found to be increased by treatments in fibroblasts compared to cells under anti-inflammatory conditions, but did not reach the levels of controls under optimal conditions (Figure 14C), despite the observed improved scratch healing (Figure 14B). *NFKB1* was increased at 6 h under anti-inflammatory conditions in keratinocytes after treatment with wIRA and heating *w*/*o* wIRA (Figure 13C). In fibroblasts, *NFKB* was found to be significantly increased at 12 h after treatments (*p* < 0.01; Figure 14C). Increased *HSP90AA1* and *HSPD1* transcript amounts in keratinocytes under anti-inflammatory settings were reduced by wIRA treatment at 12 h, reaching statistical significance for *HSP90AA1*. *HSPA1A* expression was increased by heating *w*/*o* wIRA (Figure 13C). Treatments were also found to significantly increase *HSPA1A* levels (*P* < 0.05) and *HSP90AA1* transcription (*p* < 0.01) in fibroblasts (Figure 14C), possibly indicating a temperature effect on the cells. *HSPD1* was increased in fibroblasts under anti-inflammatory conditions compared to the control and not affected by treatments. Treatments significantly increased keratinocyte *DSG1* levels (*p* < 0.1) in correlation with their effectiveness to enhance scratch closure, but to a lesser extent compared to normal conditions. *DSG3* levels were in contrast not enhanced, but rather decreased despite the positive effect on wound healing progression (Figure 13C). Treatments did not significantly affect *S100A7* levels under anti-inflammatory conditions and *RNASE7* transcription was even reduced. However, *DEFB1* gene expression was significantly augmented after heating *w*/*o* wIRA as well as to a lesser extent after wIRA radiation (Figure 13C), coinciding with the improved scratch closure (Figure 13B). Furthermore, radiation with wIRA, but not heating *w*/*o* wIRA increased *COL1A1* and *COL3A1* transcript levels in fibroblasts distinctly (Figure 14C).

## 4. Discussion

Photobiomodulation has been shown to exert beneficial effects on wound healing [26,27], matrix deposition, and cell layers [29,30,90,91]. Significant induction of wound healing processes in acute and chronic wounds by wIRA treatment have been reported [26,27]. Treatment with wIRA radiation improved scratch healing in vitro. While the influence on keratinocytes seems mostly based on a warming effect [92], fibroblasts appear to be directly stimulated by the wIRA radiation. In contrast, UV-B irradiation resulted in severe damage to keratinocytes and fibroblasts and scratch wounds showed no tendency for healing. Positive in vitro effects of wIRA have been previously described [32,33,93]. However, differences between the in vivo and the experimental in vitro conditions exist as chronic wounds, for instance, are characterized by microbial infection [5,6] and continuous conditions of inflammation [7], stalling the healing process. Moreover, TH2-dominant, anti-inflammatory conditions have been reported to affect wound healing processes [8,9,10]. The experimental prerequisites in the scratch assays applied so far accounted for optimal cell conditions. Hence, the study was extended to include the readjustment of specific milieus, more closely mimicking different detrimental in vivo situations, explicitly *S. aureus* infection, chronic inflammation as well as anti-inflammatory conditions. Scratch wound healing progression under all non-optimal environments was found to be significantly reduced. The influence was most pronounced for the infection with *S. aureus*, which damaged the cells to an extent that scratch wounds did not heal in vitro. *S. aureus* is known to induce cell death in keratinocytes in a co-culture model for infection [94]. In accordance, no living cells were noted after 24 and 48 h under infection conditions. Under anti-inflammatory conditions, where keratinocytes and fibroblasts were primed with IL-4 and IL-13 for 24 h prior to scratching, scratch wound healing was also significantly impeded. IL-4 and IL-13 are pleiotropic cytokines involved in cell growth, immune system regulation, induction of anti-inflammatory process, and M2 macrophage promotion [8]. Thus, they are thought to contribute to the wound healing process by stimulation of the repair process [10]. However, they have also been implicated in the pathogenesis of fibroproliferative disorders such as hypertrophic scar formation or systemic sclerosis [8,95]. However, in this study, reduced keratinocyte and fibroblast scratch healing was observed, which is in accordance with results obtained by Serezani et al., demonstrating impaired wound healing in keratinocytes stimulated with IL-4 [9] and consistent with the decreased wound repair found in atopic children [42]. Chronic inflammatory conditions, produced by priming with TNF-α and IFN-γ [41], also reduced scratch healing compared to the controls under optimal conditions mainly by delaying cell layer regeneration. Persistence of inflammation is detrimental and can cause cell damage, thus stalling the healing process [4]. It was found that wIRA treatment improved keratinocyte and fibroblast scratch healing under chronic inflammatory conditions beyond the extent of cells under optimal conditions. The effect was only partly dependent on warming alone and could be an explanation for the positive effects observed in chronic wounds [26]. Moreover, a distinct improvement of the wound healing progression in keratinocytes and fibroblasts under anti-inflammatory conditions was achieved by wIRA radiation, significantly enhancing scratch closure.

Layer repair in the keratinocyte and fibroblast wound healing models was assessed by gene expression analysis of typical proteins in epidermis and dermis. The keratinocytes form a tight cell barrier by connecting neighboring cells to each other through intercellular junctions, which include (corneo) desmosomes, adherens junctions, and tight junctions [88]. Desmosomes are composed of desmosomal cadherins such as desmogleins (Dsg) 1–4 and desmocollins 1–3, which are transmembrane glycoproteins of the cadherin superfamily [89]. *DSG1* and *DSG3* gene expression by keratinocytes increased over time during scratch wound healing, which is consistent with the regeneration of the epithelial layer and the re-formation of cell–cell-contacts [96]. To allow wound healing, the cell adhesion proteins need to remodel, first detaching to enable cell migration and subsequently reforming to reestablish the paracellular barrier in the skin [97,98]. Transcript levels of the cell adhesion molecule genes *DSG1* and *DSG3* were found to be increased after wIRA treatment compared to the control corresponding to the differences observed in the healing progression over time. Significantly reduced amounts were observed after UV-B irradiation as well as under non-optimal conditions. Treatment with wIRA restored *DSG1* levels under chronic inflammatory to levels under optimal conditions and significantly increased *DSG1* levels under anti-inflammatory conditions in correlation with the improvement in scratch wound closure. Regeneration of the dermal cell layer in this in vitro model was assessed by collagen gene transcription in fibroblasts. *COL1A1* and *COL3A1* levels were found to be significantly increased over time corresponding to the reformation of the fibroblast cell layer. Gene expression of *COL3A1* was especially found to be significantly augmented after wIRA treatments corresponding to the healing progression observed. Interestingly, the most pronounced increase in the transcript levels of these genes was found after UV-B irradiation in the fibroblasts. So far, in unscratched fibroblasts, a reduction in the expression of *COL1A1* and *COL3A1* after UV-B irradiation was observed [99,100]. UV light is known to have an adverse effect on dermal collagen either by direct degradation through increased MMP production [101] or by inhibiting pro-collagen biosynthesis [102] as well as reducing collagen gene expression [99,100]. These processes have been strongly linked to the formation of wrinkles induced by the augmentation of oxidative stress by UV light affecting several downstream events of collagen metabolism, making UV light one of the most powerful physical agents responsible for extrinsic skin aging [103]. However, UV-B exposure has also been reported to exacerbate and prolong the corneal stromal healing response manifesting as augmented sub-epithelial haze and deposition of disorganized collagen [104]. *COL1A1* and *COL3A1* gene expression was affected by chronic inflammatory conditions, possibly corresponding to the increase in *IL1A* and *NFKB1* levels and was found to be significantly reduced after anti-inflammatory conditioning with IL-4/IL-13. In both cases, radiation with wIRA markedly enhanced *COL1A1* and *COL3A1* transcription, reestablishing control transcript levels or even exceeding those, reflecting the improved scratch wound healing progression.

From the results obtained in the wound healing studies with keratinocytes and fibroblasts under infection conditions, it was concluded that wIRA does not convey an antimicrobial activity. However, wIRA has been shown to exert antibacterial effects on bacteria that are either heat-sensitive [105] or when used in combination with photosensitizers as photodynamic therapy [106,107]. However, previous studies have also failed to note direct antimicrobial effects of wIRA on wound pathogens [108], which can be explained by the fact that these wound pathogens are not heat-sensitive. Treatment with wIRA slightly stalled the negative effects of *S. aureus* and keratinocytes remained viable up to 24 h in contrast to untreated keratinocytes, but no protective effect on fibroblasts was observed. It was hence presumed that the better survival of infected keratinocytes after wIRA treatment could depend on the induction of antimicrobial peptides (AMPs) such as psoriasin and defensin by keratinocytes. As innate immune cells, keratinocytes can produce a wide variety of AMPs upon stimulation [34,76], which are important effector molecules with broad spectrum anti-microbial activity against bacteria, fungi, and even viruses [77,78,79,80,81].

AMP gene expression increased during scratch wound healing. Endogenous AMPs have also been found to be upregulated in all stages of wound healing in vivo, indicating a role beyond microbial defense toward regulation of immune response, granulation tissue formation, and re-epithelization [82,83]. It was also observed that antimicrobial peptide transcript levels were significantly elevated after wIRA treatment under optimal conditions, further suggesting the involvement in the improved scratch wound healing outcome. However, the observed increase in *S100A7* and *RNASE7* transcription after UV-B irradiation was probably due to stimulation by the elicited inflammatory response [109]. Expression of the AMP genes *S100A7* and *RNASE7* was also significantly induced by *S. aureus* infection at early time points. *S100A7* transcription levels were not affected by chronic inflammatory conditions while *RNASE7* levels were slightly decreased and *DEFB1* was significantly reduced. Insufficient upregulation of defensins in chronic wounds such as diabetic foot and venous leg ulcers has been suggested to contribute to the chronicity of ulcers by reduced antimicrobial defense [84,85]. Here, it was found that *DEFB1* gene expression was significantly increased after wIRA radiation under chronic inflammatory conditions. Although it did not reach expression levels under optimal conditions during scratch wound healing progression, it most likely confers a beneficial influence. Hence, induction of defensins might contribute to the positive effects of wIRA radiation reported in chronic wounds [26]. Moreover, levels of *S100A7*, *RNASE7*, and *DEFB1* were generally lower under anti-inflammatory conditions compared to optimal conditions. Recent studies indicate that an increased TH2 cytokine expression contributes to the reduction in AMPs observed in atopic dermatitis, resulting in frequent skin infections [86,87]. While wIRA treatment did not increase *S100A7* and *RNASE7* levels, *DEFB1* transcription was augmented in correspondence with the improved scratch closure. In accordance, wIRA could have a potential positive influence on frequent skin infections of patients with atopic dermatitis [86,87] by boosting skin defense mechanisms. However, contrary to expectations after improved survival of infected keratinocytes, wIRA radiation significantly decreased AMP gene transcription under infection conditions. Hence, it is unlikely that an augmented production of AMPs conferred the resistance to *S. aureus* infection under wIRA treatment.

Keratinocytes and fibroblasts secrete a broad spectrum of cytokines, chemokines, and growth factors including IL-1α, IL-6, IL-8, TGF-β, and PDGF [34,35], all with a possible role in wound healing and therefore potential targets for photobiomodulation by wIRA. Radiation with wIRA did not induce transcription of pro-inflammatory cytokine genes compared to the untreated control under optimal conditions, while UV-B irradiation was found to elicit a detrimental inflammatory response in keratinocytes and fibroblasts. Additionally, bacteria and the formation of biofilms can drive inflammation by interaction with the wound cells [7]. Accordingly, gene expression analysis revealed an early and significant induction of *IL1A*, *IL6*, and *CXCL8* expression during infection of keratinocytes with *S. aureus* accounting for the detrimental effects observed. However, infection with *S. aureus* failed to elicit pro-inflammatory cytokine gene expression in fibroblasts. The fibroblasts rapidly died after the infection with *S. aureus*, most likely due to the damaging effects of *S. aureus* (e.g., via the release of toxins). This may also occur due to starving of the cells as bacteria take up the nutrients in the medium. As expected, a chronic inflammatory milieu led to increased *IL1A* mRNA levels compared to the control while anti-inflammatory conditions reduced *IL1A* transcription. Acting as the “first alarm system” of the skin, the IL-1 cytokine family plays a crucial role through alerting the body to immediate dangers and initiating the inflammatory cascade in the skin as well as inducing gene expression and synthesis of other inflammatory mediators [45,46]. *IL6* gene expression showed no distinct differences to the control under chronic inflammatory conditions, while *CXCL8* levels were neither affected in keratinocytes, a significant decrease in fibroblasts was found. Surprisingly, *IL-6* gene expression was induced in fibroblasts by the combination of IL-4 and IL-13. Treatment with wIRA had the potential to decrease pro-inflammatory cytokine gene expression in infected keratinocytes as well as under chronic inflammatory conditions. In general, anti-inflammatory therapies have been associated with a positive influence on the wound healing outcomes due to the moderating effects on inciting cytokines and reactive oxygen species [110,111,112]. However, the initial inflammatory response after injury is essential for wound healing and stimulates regenerative processes [41]. The promotion of the acute inflammation can increase wound healing [39,47] and the reduction in IL-6 by anti-inflammatory treatment has been shown to inhibit scratch wound healing [48]. In accordance, scratch wound healing after IL-4/IL-13 stimulation was found to be decreased and could be re-initiated by wIRA treatment, which induced *IL1A* gene expression under anti-inflammatory conditions at one hour. Similarly, low-level laser therapy and cold atmospheric plasma were able to induce IL-8 secretion and increase wound healing in vitro [90,113].

Photobiomodulation has been demonstrated to promote the production of growth factors such as PDGF, TGF, and bFGF as well as activate the mitogenic signaling pathway in fibroblasts [91]. TGF-β plays an important role in wound healing by stimulating granulation tissue formation [49,50,51,52] and mediating fibroblast proliferation, collagen production, ECM deposition and myofibroblast differentiation [53,54,55] as well as promoting angiogenesis [1]. Hence, increased TGF-β activity can accelerate wound healing [56,57] and positively impact the production of dermal-epidermal junction proteins [114]. Induction of *TGFB1* has also been associated with the promotion of keratinocyte proliferation and migration [58]. Here, it was observed that *TGFB1* transcript levels significantly increased over time in keratinocytes and showed a peak as early as 12 h in fibroblasts. With the onset of an injury toward healing, expression levels have been shown to be increased [115,116,117]. A slight surge of *PDGFC* gene expression was observed for keratinocytes at 3 h with a subsequent decrease while fibroblasts demonstrated a steady rise in the gene’s expression over time under these conditions. PDGF-C is a key component of the PDGFR-α signaling pathway and possesses a similar modulation capacity of fibroblast differentiation as TGF-β [59]. *TGFB1* and *PDGFC* gene expression increased under chronic inflammatory conditions over time, corresponding to scratch wound healing. However, the response in fibroblasts was significantly decreased compared to optimal conditions reflecting the slower progression. Overexpression may also contribute to abundant collagen accumulation, tissue fibrosis, and pathological scar formation [8,118,119], making it a double-edged sword. Significantly decreased *TGFB1* or *PDGFC* levels were observed under anti-inflammatory conditions in fibroblasts, probably accounting for the reduced scratch healing found here. *TGFB1* levels were also slightly reduced in keratinocytes under anti-inflammatory conditions. Treatment with wIRA significantly increased *TGFB1* levels in keratinocytes in the chronic inflammatory milieu and induced *TGFB1* as well as *PDGFC* gene expression under anti-inflammatory conditions in keratinocytes and fibroblasts. In addition, transcription factors play a crucial role in stress response, damage control, and wound healing. The NF-κB transcription factors have been implicated in the regulation of several genes, thus governing many biological effects including apoptosis, immune response, and inflammatory processes [60,61,62]. It has previously been shown that NF-κB is a major regulator of cell proliferation and migration, acting as a transcription factor for both cyclin D and MMP-9 [63], and is essential for wound healing [64]. Similarly, p53 (gene *TP53*) regulates cell cycle progression after DNA damage, activation of DNA repair mechanisms, and cell apoptosis, if DNA damage proves to be irreparable. Correspondingly, the gene expression of *TP53* and *NFKB* was elevated during wound healing [63,64]. Here, *TP53* levels were significantly increased by infection of keratinocytes with *S. aureus* and in fibroblasts under chronic inflammatory conditions as well as wIRA radiation under optimal conditions. Chronic and anti-inflammatory conditions also significantly induced *NFKB1* in fibroblasts. *TP53* levels were not affected by UV-B irradiation, although protein levels of p53 in keratinocytes and fibroblasts have been reported to be dramatically increased after UV-B exposure, eventually resulting in cell apoptosis [120]. Under non-optimal conditions, wIRA treatment was found to significantly reduce *TP53* levels in keratinocytes during infection and in fibroblasts under chronic inflammation. In contrast, *NFKB1* levels were elevated in wIRA-treated, infected keratinocytes and wIRA radiation also induced *NFKB1* gene expression in keratinocytes and fibroblasts under anti-inflammatory conditions. The fostered activation of NF-κB has been shown to improve wound healing in vitro [121], which could account for the observed scratch healing induction. However, *NFKB1* was significantly reduced in TNF-α/IFN-γ-primed cells through wIRA treatment, despite showing an equal improvement in scratch wound healing.

Heat shock proteins (HSPs) have not been studied extensively so far in scratch wound healing models. They constitute a large group of chaperone proteins found in virtually all organisms where they modulate cellular homeostasis, aid in repair after cellular stress, and promote wound healing [47]. HSPs are classified into several families on the basis of their molecular size and amino acid sequence similarity with highly differentiated expression patterns, intracellular localization, and function [68]. They are induced under conditions of cell stress (e.g., inflammation) [68]. For instance, HSP90 is an abundant cellular protein constituting about 1–2% of total protein in non-stressed cells and about 4–6% in stressed tissues [69,70]. It assists in proper protein folding and prevents aggregation of non-native proteins [71]. The HSPA1 protein (previously designated HSP70) is another cytoprotective agent with very low transcript levels in unstressed normal cells, where it plays a crucial role in guiding conformational status of the proteins during folding and translocation [72] and conferring thermo-resistance [73]. HSPD1 (previously HSP60) is foremost located as a chaperonin in the cytosol and mitochondrial matrix [74]. *HSPA1A* and *HSPD1* genes were more strongly expressed in cell layers after scratch wounding and during scratch healing progression, highlighting their role as chaperones in protein folding and as possible cell regulators [65,66,67]. HSPD1 has also been shown to induce an inflammatory response through several different receptors including TLR2, TLR4, and CD36 [65,67]. Consequently, it is expected that HSPs can further act as potent immune activators outside of cells, where they induce various pro-inflammatory cytokines, interact with antigenic polypeptides, and assist in antigen presentation [66]. *HSP90AA1* and *HSPD1* levels were slightly increased by UV-B irradiation in keratinocytes. However, transcription of *HSPA1A* was not altered after UV-B irradiation in keratinocytes, but was significantly induced by heating *w*/*o* wIRA under optimal conditions. HSPA1 activation indicates the condition of cellular stress [122], which is not limited to heat shock, but can also range from oxidative stress to ischemia and inflammation or aging [123]. UV-B irradiation also induced *HSP90AA1*, *HSPA1A*, and *HSPD1* gene expression in fibroblasts and these HSPs might therefore be implicated in the inhibition of scratch healing. Stress response gene transcription level evaluation further pointed to condition-specific increases in the HSPs’ gene expression under infection as well as chronic and anti-inflammatory conditions. TNF-α/IFN-γ stimulation resulted in an increase in *HSPD1* and *HSP90AA1* gene expression in both cell types as well as a significant elevation in *HSPA1A* levels in fibroblasts. *S. aureus* notably stimulated *HSPA1A* and *HSP90AA1* gene expression in keratinocytes, but not in fibroblasts. Anti-inflammatory conditions led to significantly higher *HSPD1* transcript levels in the cell scratches compared to the control. In keratinocytes, this was also found for *HSP90AA1.* It is most likely that non-optimal conditions affect HSP gene expression due to their role as chaperones in protein folding and possible cell regulators [65,66,67]. Under infection, wIRA radiation could restore *HSPA1A* transcription to control levels and alleviate *HSP90AA1* increases in keratinocytes. This might contribute to the prolonged survival of wIRA-treated keratinocytes under infection conditions. For instance, HSF1 is known to initiate host defense against bacterial infection, partly through promoting early TLR2 signaling activation [124]. Treatment with wIRA had no significant effect in infected fibroblasts. Hence, it seems likely that toxic effects of the bacteria are overwhelming the natural cellular defense mechanism and impede potential protective and regulatory events, resulting in the abolished scratch closure. On the other hand, HSPs have been associated with the internalization of *S. aureus* and their inhibition was implicated in the protection against bacterial infection [125]. *HSPD1* and *HSPA1A* transcription have been found to be significantly augmented by wIRA radiation in keratinocytes under chronic inflammatory conditions. HSDP1 has been implicated in wound healing [75] and might therefore confer the improved wound healing observed under these conditions after wIRA treatment. However, the same gene transcripts were reduced in fibroblasts to control levels, which might be another explanation as to why wIRA radiation exhibits a beneficial effect in fibroblast layer regeneration after scratching. Moreover, under anti-inflammatory settings, the increased gene expression levels of *HSP90AA1* and *HSPD1* were reduced by wIRA treatment in keratinocytes and in contrast, wIRA radiation induced *HSPA1A* and *HSP90AA1* transcription in fibroblasts. Hence, further research is warranted to illuminate the effect of photobiomodulation on HSPs and their role in wound healing after wIRA treatment.

## 5. Conclusions

Keratinocyte and fibroblast wound healing under all non-optimal environments was found to be distinctly reduced in vitro. The influence was most pronounced for the infection with *S. aureus*, which damaged the cells to an extent that scratch wounds did not heal. Under chronic inflammatory and anti-inflammatory conditions, scratch wound healing was also significantly impeded, which was considerably improved by treatment with wIRA. The regenerative effect of wIRA radiation under non-optimal conditions could be an explanation for the positive effects observed previously in chronic wounds [26]. The enhancing effect of wIRA treatment on scratch wound closure was confirmed by a slight increase in *DSG1* as well as a significant induction of *DSG3* levels in keratinocytes under optimal conditions. Furthermore, wIRA was able to restore *DSG1* and *DSG3* transcript levels under chronic inflammatory conditions to these control levels and elicited a significant increase in *DSG1* gene expression under anti-inflammatory conditions. In addition, wIRA especially induced gene expression of *COL3A1* under all conditions and to some extent, promoted *COL1A1* transcription under chronic and anti-inflammatory conditions in fibroblasts. It was also found that wIRA treatment had the potential to counteract the inflammatory response in infected keratinocytes as well as under chronic inflammatory conditions by decreasing pro-inflammatory cytokine gene expression. In contrast, in the anti-inflammatory setting, wIRA radiation could re-initiate the acute inflammatory response, which has been found necessary after injury to stimulate regenerative processes [41] by increasing *IL1A* levels in keratinocytes and early transcription of *IL1A*, *IL6*, and *CXCL8* in fibroblasts. These results were further confirmed by observed increases of *TGFB1* levels in wIRA-treated cells under chronic and anti-inflammatory conditions as well as induction of *PDGFC* in the latter case. Positive effects of wIRA could also partly be linked to elevation of *NFKB1* levels under anti-inflammatory conditions as NF-κB has been shown to improve wound healing in vitro [121]. Most interesting were the results for the expression of heat shock protein genes, which have not been studied extensively so far in scratch wound healing models. *HSPA1A* and *HSPD1* were differently transcribed in cell layers after scratch wounding and during scratch healing progression. Non-optimal conditions promoted increased transcript levels. Under infection, wIRA radiation could restore *HSPA1A* levels to control levels and alleviate *HSP90AA1* increases in keratinocytes, which might have contributed to the slightly improved survival of wIRA-treated, infected keratinocytes. Furthermore, *HSPD1*, which has been previously implicated in wound healing [1], and *HSPA1A* gene expression have been found to be significantly augmented by wIRA radiation in keratinocytes under chronic inflammatory conditions. However, contradicting results have been found between keratinocytes and fibroblasts. Hence, further research seems warranted to illuminate the effect of photobiomodulation on HSPs and their role in wound healing after wIRA treatment.

## Figures and Tables

**Table 1:** Overview of the biological functions of the examined target genes.

Grouping	Target Gene	Biological Function
Inflammatory cytokines	*IL1A*	- keratinocytes and fibroblasts secrete a broad spectrum of cytokines including IL-1α, IL-6, and IL-8 [34,35]; - the IL-1 cytokine family plays a crucial role through alerting the body to immediate dangers and initiating the inflammatory cascade in the skin as well as inducing gene expression and synthesis of other inflammatory mediators [45,46]; - the initial inflammatory response after injury is essential for wound healing and stimulates regenerative processes [41]; - promotion of the acute inflammation can increase wound healing [39,47]; and - reduction in IL-6 by anti-inflammatory treatment has been shown to inhibit scratch wound healing [48].
	*IL6*	
	*CXCL8*	
Growth factors	*TGFB1*	- keratinocytes and fibroblasts secrete a broad spectrum of growth factors including TGF-β and PDGF [34,35]; - TGF-β plays an important role in wound healing (stimulating granulation tissue formation [49,50,51,52], mediating fibroblast proliferation as well as collagen production, ECM deposition and myofibroblast differentiation [53,54,55]); - TGF-β promotes angiogenesis [1]; - increased TGF-β activity can accelerate wound healing [56,57]; - induction of TGFB1 has been associated with promotion of keratinocyte proliferation and migration [58]; and - PDGF-C is a key component of the PDGFR-α signaling pathway and possesses similar modulation capacity of fibroblast differentiation as TGF-β [59].
	*PDGFC*	
Transcription factors	*NFKB1*	- NF-κB transcription factors have been implicated in the regulation of several genes: apoptosis, immune response and inflammatory processes [60,61,62]; - NF-κB is a major regulator of cell proliferation and migration acting as transcription factor for both, cyclin D and MMP-9 [63]; - NF-κB is essential for wound healing [64]; - p53 (gene TP53) regulates cell cycle progression after DNA damage, activation of DNA repair mechanisms and cell apoptosis, if DNA damage proves to be irreparable [64]; and - expression of TP53 and NFKB is elevated during wound healing [63,64].
	*TP53*	
Heat shock proteins	*HSP90AA1*	- HSPs have crucial roles in protein folding and as possible signaling regulators inducing cellular stress responses [65,66,67]; - constitute a large group of chaperone proteins found in virtually all organisms, where they modulate cellular homeostasis, aid in repair after cellular stress and promote wound healing [47]; - classified into several families on the basis of their molecular size and amino acid sequence similarity with highly differentiated expression patterns, intracellular localization and function [68]; - induced under conditions of cell stress, e.g., inflammation [68]; - HSP90 is an abundant cellular protein constituting about 1–2% of total protein in non-stressed cells and about 4–6% in stressed tissues [69,70]; - HSP90 assists proper protein folding and prevents aggregation of non-native proteins [71]; - HSPA1 (previously designated HSP70) is another cytoprotective agent with very low transcript levels in unstressed normal cells; - HSPA1 plays a crucial role in guiding conformational status of the proteins during folding and translocation [72] and conferring thermo-resistance [73]; - HSPD1 (previously HSP60) is foremost located as a chaperonin in the cytosol and mitochondrial matrix [74]; - HSPD1 induces an inflammatory response through several different receptors including TLR2, TLR4 and CD36 [65,67]; and - HSDP1 has been implicated in wound healing [75].
	*HSPA1A*	
	*HSPD1*	
Antimicrobial peptides	*DEFB1*	- keratinocytes can produce a wide variety of AMPs upon stimulation [34,76]; - AMPs are important effector molecules with broad spectrum anti-microbial activity against bacteria, fungi and even viruses [77,78,79,80,81]; - endogenous AMPs are upregulated in all stages of wound healing in vivo indicating a role beyond microbial defense toward regulation of immune response, granulation tissue formation and re-epithelization [82,83]; - insufficient upregulation of defensins in chronic wounds such as diabetic foot and venous leg ulcers has been suggested to contribute to the chronicity of ulcers by reduced antimicrobial defense [84,85]; and - an increased TH2 cytokine expression contributes to the reduction in AMPs observed in atopic dermatitis resulting in frequent skin infections [86,87].
	*RNASE7*	
	*S100A7*	
Structural components	*DSG1*	- keratinocytes form a tight cell barrier by connecting neighboring cells to each other through intercellular junctions, which include (corneo) desmosomes, adherens junctions and tight junctions [88]; and - desmosomes are composed of the desmosomal cadherins such as desmogleins (Dsg) 1–4 and desmocollins 1–3, which are transmembrane glycoproteins of the cadherin superfamily [89].
	*DSG3*	
	*COL1A1*	- collagen, type I, alpha 1, also known as alpha-1 type I collagen; - is the major component of type I collagen, the fibrillar collagen found in most connective tissues including cartilage; - type III collagen is found in the skin, lungs, intestinal walls, and the walls of blood vessels; and - components of type III collagen, called pro-α1(III) chains, are produced from the COL3A1 gene.
	*COL1A3*	

**Table 2:** List of primer sequences and ordering IDs used for the SYBR Green-based RTqPCR.

Target Gene	Ordering IDs/Primer Sequence		Manufacturer
*ACTB*	Hs_ACTB_1_SG QuantiTect^®^ Primer Assay		Qiagen, Hilden, Germany
*CXCL8*	Hs_CXCL8_1_SG QuantiTect^®^ Primer Assay		
*TGFB1*	Hs_TGFB1_1_SG QuantiTect^®^ Primer Assay		
*PDGFC*	Hs_PDGFC_1_SG QuantiTect^®^ Primer Assay		
*RNASE7*	Hs_RNASE7_1_SG QuantiTect^®^ Primer Assay		
*COL1A1*	Hs_COL1A1_1_SG QuantiTect^®^ Primer Assay		
*COL1A3*	Hs_COL1A3_1_SG QuantiTect^®^ Primer Assay		
*HSP90AA1*	QT01002603		
*HSPA1A*	QT01002568		
*HSPD1*	QT00018970		
*NFKB1*	QT00063791		
*TP53*	QT00060235		
*DEFB1*	QT00008302		
*IL1A*	Fw Rev	5′-CGCCAATGACTCAGAGGAAGA-3′ 5′-AGGGCGTCATTCAGGATGAA-3′	Eurofins Genomics, Ebersberg, Germany
*IL6*	Fw Rev	5′-CCACCGGGAACGAAAGAGAA-3′ 5′-GAGAAGGCAACTGGACCGAA-3′	
*S100A7*	Fw Rev	5′-GTCCAAACACACACATCTCACT-3′ 5′-TCATCATCGTCAGCAGGCTT-3′	
*DSG1*	Fw Rev	5′-TCCCCACATTTCGGCACTAC-3′ 5′-GCCCAGAGGATCGAGAATAGG-3′	
*DSG3*	Fw Rev	5′-GTCAGAACAATCGGTGTGAGATG-3′ 5′-TGCGGCCTGCCATACCT-3′	

## Data Availability

The data in this study are available on request from the corresponding author.

## Abbreviations

AMP	antimicrobial peptide
COL	collagen
CXCL	C-X-C motif ligand
DSG	desmoglein
ELISA	enzyme linked immunosorbent assay
HSP	heat shock protein
IL	interleukin
IFN	interferon
MMP	matrix metalloproteinase
n.d.	not determined
NFKB	nuclear factor ‘kappa-light-chain-enhancer’ of activated B-cells
PDGF	platelet derived growth factor
qPCR	quantitative polymerase chain reaction
ROS	reactive oxygen species
TGF	transforming growth factor
TNF	tumor necrosis factor
TP	tumor suppressor gene
UV-B	ultraviolet light B
*w*/*o*	without
wIRA	water-filtered infrared A
